# An Integrated Assessment Model for Helping the United States Sea Scallop (*Placopecten magellanicus*) Fishery Plan Ahead for Ocean Acidification and Warming

**DOI:** 10.1371/journal.pone.0124145

**Published:** 2015-05-06

**Authors:** Sarah R. Cooley, Jennie E. Rheuban, Deborah R. Hart, Victoria Luu, David M. Glover, Jonathan A. Hare, Scott C. Doney

**Affiliations:** 1 Ocean Conservancy, Washington, DC, United States of America; 2 Department of Marine Chemistry and Geochemistry, Woods Hole Oceanographic Institution, Woods Hole, Massachusetts, United States of America; 3 NOAA NMFS NEFSC Woods Hole Laboratory, Woods Hole, Massachusetts, United States of America; 4 Department of Earth and Environmental Sciences, Boston College, Chestnut Hill, Massachusetts, United States of America; 5 NOAA NMFS NEFSC Narragansett Laboratory, Narragansett, Rhode Island, United States of America; University of Gothenburg, SWEDEN

## Abstract

Ocean acidification, the progressive change in ocean chemistry caused by uptake of atmospheric CO_2_, is likely to affect some marine resources negatively, including shellfish. The Atlantic sea scallop (*Placopecten magellanicus*) supports one of the most economically important single-species commercial fisheries in the United States. Careful management appears to be the most powerful short-term factor affecting scallop populations, but in the coming decades scallops will be increasingly influenced by global environmental changes such as ocean warming and ocean acidification. In this paper, we describe an integrated assessment model (IAM) that numerically simulates oceanographic, population dynamic, and socioeconomic relationships for the U.S. commercial sea scallop fishery. Our primary goal is to enrich resource management deliberations by offering both short- and long-term insight into the system and generating detailed policy-relevant information about the relative effects of ocean acidification, temperature rise, fishing pressure, and socioeconomic factors on the fishery using a simplified model system. Starting with relationships and data used now for sea scallop fishery management, the model adds socioeconomic decision making based on static economic theory and includes ocean biogeochemical change resulting from CO_2_ emissions. The model skillfully reproduces scallop population dynamics, market dynamics, and seawater carbonate chemistry since 2000. It indicates sea scallop harvests could decline substantially by 2050 under RCP 8.5 CO_2_ emissions and current harvest rules, assuming that ocean acidification affects *P*. *magellanicus* by decreasing recruitment and slowing growth, and that ocean warming increases growth. Future work will explore different economic and management scenarios and test how potential impacts of ocean acidification on other scallop biological parameters may influence the social-ecological system. Future empirical work on the effect of ocean acidification on sea scallops is also needed.

## Introduction

Fisheries science has long incorporated the effects of fishing, a human perturbation, into marine harvest advice as well as short and long-term forecasts (e.g., [1]). However, including the effects of other anthropogenically driven global changes, like warming and changing water chemistry, is relatively new [2–7]. Additionally, socioeconomic factors influence marine policy and management significantly, yet they have been traditionally given less attention and separate treatment [8]. One reason for this relatively slow incorporation of global change into fisheries advice is that it remains challenging to distinguish the effects of climate, particularly temperature rise, from those of fishing and natural environmental or biological stochasticity in many contexts [9], and references therein). Another reason is the different timescales of concern: fisheries plans are developed every few years, whereas effects of global change occur progressively over decades. Planning for present and future fishery harvests against a background of global change, though, requires understanding how both short- and long-term processes associated with environmental and social factors affect the fishery through direct and indirect routes [10]. Integrated assessment models (IAMs) that numerically simulate oceanographic, population dynamic, and socioeconomic relationships provide one way to bring together these disciplines and provide both short- and long-term perspectives on how different components of the social-ecological system affect each other. In their simplest forms, IAMs can be developed for single-species fisheries that are subject to a limited set of major environmental and human influences and whose socioeconomic importance is relatively easy to quantify monetarily.

### Atlantic sea scallop

Atlantic sea scallops (*Placopecten magellanicus*) support one of the most economically important single-species fisheries in the United States. They are found along the eastern North American continental shelf from Virginia to the Gulf of Maine and north into Canada, but primary harvest areas in the U.S. are located in the Mid-Atlantic Bight and Georges Bank, between 35 and 100m depth [11]. A small Gulf of Maine population is in nearshore, shallow, state waters and comprises only a few percent of the annual harvest each year [12]. Most sea scallops are typically found in waters that remain below 17°C and above 0°C, although they can tolerate temperatures as high as 20°C [11]. Larvae remain planktonic in the upper water column for the first 4–7 weeks of life before settling to the bottom. Scallops become sexually mature at around age 2, reach a commercially harvestable size at about age 4 (90 mm), and can reach ages of at least 18–20 years, with a maximal shell height ranging from 125–180 mm [13,14]. Populations living in the Mid-Atlantic and Georges Bank regions have slightly different growth parameters; the Mid-Atlantic population reaches a lower average maximum size, but at a slightly faster rate, compared to that farther north [14].

**Fig 1:**
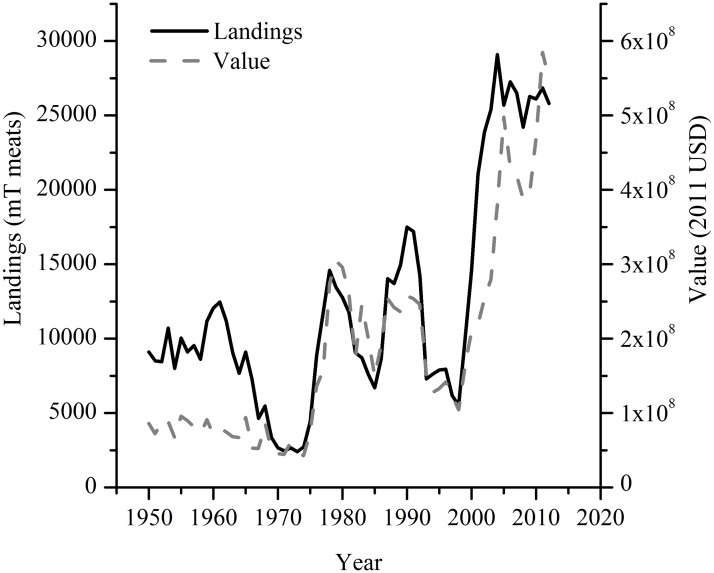
US sea scallop harvests through time (NMFS commercial harvest data accessed January 28, 2014). Values are adjusted to 2011 US dollars.

The sea scallop fishery, which grossed $559 million in dockside revenue in 2012 [15] (Fig 1), is intensively managed with an area rotational management scheme that includes limited permits and restrictions on days at sea, gear size, and crew size [16]. Regular population surveys inform the spatial area management simulator (SAMS) and a catch-at-size-analysis (CASA) model [12,17], which provide information used in the determination of allowable catch limits. Although careful management appears to be the most powerful short-term factor that affects scallop populations [16], scallops will be increasingly influenced by long-term global environmental change in the coming decades as bottom temperatures rise [18] and ocean acidification advances.

### Ocean acidification

Ocean acidification refers to a series of chemical changes caused by the uptake of CO_2_ into seawater: elevated aqueous CO_2_ and total inorganic carbon, and reduced pH, carbonate ion, and calcium carbonate mineral saturation states [19]. Fossil fuel combustion and deforestation have caused global mean atmospheric CO_2_ levels to grow by 40% from about 280 ppm in the preindustrial era to 396 ppm by 2013 (www.esrl.noaa.gov/gmd/ccgg/trends/), causing a net global air-to-sea flux of excess CO_2_ that dissolves about 25% of total anthropogenic carbon emissions in seawater [20]. Consequently, sea-surface pH is estimated to have dropped by 0.1 pH units since the preindustrial era, a 26% increase in acidity over the past 150 years, mostly in the past several decades. Future projections suggest declines of an additional 0.2–0.3 pH units over this century [21]. At the same time, global sea surface temperatures have increased by 0.8°C with substantial regional variability [22].

On the continental shelf in the Mid-Atlantic Bight and Georges Bank, mean summertime sea surface saturation state of aragonite (Ω_ar_), a more soluble mineral form of calcium carbonate and one commonly found in larval bivalve shells, declines towards the north, beginning at around Ω_ar_ = 3 east of North Carolina, decreasing to 2.5 southeast of Massachusetts, and to 1.9 east of New Hampshire [23,24]. In winter, the south to north gradient still exists, but overall levels are lower ranging from Ω_ar_ = 2.2 east of North Carolina and 1.5 east of New Hampshire [23]. The response of *P*. *magellanicus* to calcium carbonate saturation state has not been determined experimentally, but other observed species-specific responses to ocean acidification among mollusks [e.g., Eastern oyster, (*Crassostrea virginica*, *Osteridae*); hard clam (*Mercenaria mercenaria*); Atlantic bay scallop (*Argopecten irradians*, *Pectinidae*)] have been mostly neutral or negative and affect growth, survival, and development (e.g., [25–29]). The king scallop, (*P*. *maximus*), which is taxonomically close to *P*. *magellanicus* and has a very similar life history, also shows reduced growth and energy budgets under ocean acidification [30–32]. By lowering carbonate ion levels and increasing carbonate solubility, ocean acidification is thought to increase the energetic cost of calcification [33] by borrowing energy allocated to other life functions, like reproduction or immunity [34,35]. In contrast, rising water temperature tends to increase growth through increases in metabolism, up to a point where it is no longer energetically favorable to allocate energy towards growth [14,36].

### Studies connecting ocean acidification and fisheries harvests

A limited number of studies to date have examined ocean acidification’s potential to affect human communities via shellfish harvests. Cooley and Doney [37] reported that ocean acidification-driven losses of ex-vessel revenues could cost the U.S. commercial shellfish fishery a cumulative total of hundreds of millions or billions of US dollars over the next fifty years, depending on discount rate and shellfish responses. There is a similar potential for losses in allied industries. Narita et al. [38] found that global costs of negative ocean acidification-related impacts on commercial shellfish production could exceed $100 billion US annually by 2100, if demand for shellfish rises with future estimated income rise. In addition, ocean acidification could endanger food security for certain seafood-dependent nations [39]. Ocean acidification’s impacts on shellfish harvests could be very costly, but detailed policy-relevant information about the relative effects of ocean acidification, rising temperatures, fishing pressure, and socioeconomic factors on specific species has yet to be developed for most species, with a few notable exceptions [10,40]). Recently, Punt et al. [40] linked population and bioeconomic models to project ocean acidification impacts on the Alaskan king crab fishery, providing both management insight and rationale for future studies.

This paper presents a simple IAM that connects reduced-form biogeochemical, population, and economic numerical models for the Atlantic U.S. sea scallop fishery. This model allows investigation of an economically important marine resource in a dynamic system affected by environmental change, fishery policies, and biological stochasticity. A biogeochemical surface-deep box model simulates conditions for sea scallop habitats in Northeast U.S. shelf waters from the present to 2050. Oceanic conditions influence a sea scallop population dynamic model that includes biological stochasticity and realistic harvest pressure. This links to an economic model that projects revenues and costs for the commercial fishery in inflation-adjusted real dollars. This paper describes the model’s construction and presents early results based on the business-as-usual RCP 8.5 CO_2_ emissions scenario [41] and constant economic growth rates.

## Data and Methods

### Model design

**Fig 2:**
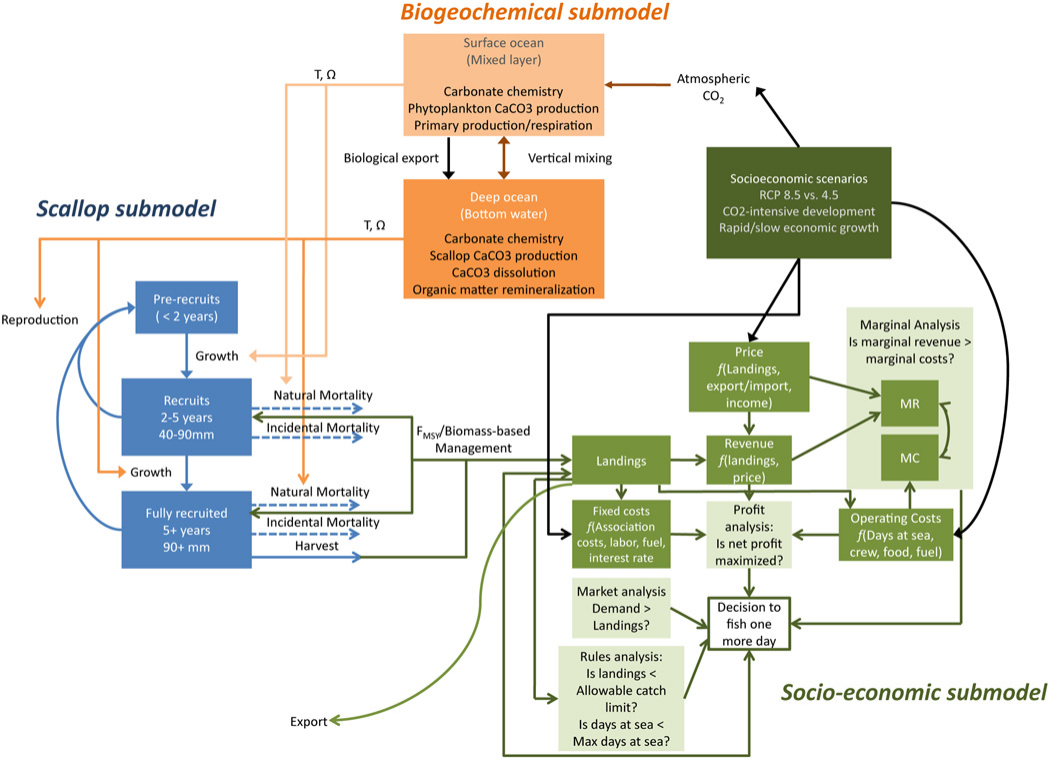
Schematic of IAM. Biogeochemical submodel components are orange, scallop submodel components are blue, and socioeconomic submodel components are green.

To build the IAM, three models were developed singly and then linked together (Fig 2). The biogeochemical model is run separately, and then used to force the scallop and socioeconomic models. The biogeochemical submodel (details in Eqs. SI1-10 in S1 Text and Table 1) is a two-box model in which temperature and salinity-driven stratification and mixing govern vertical mixing between the surface and deep (benthic) boxes on the continental shelf [42–44]. The surface—deep two-box model is solved separately for both Georges Bank (GB) and Mid Atlantic Bight (MA) regions at each time step. Carbonate chemistry is fully modeled in each box using the CO2SYS for Matlab software [45,46], including air-sea gas exchange driven by modeled temperatures (Eqs. SI1-SI6 in S1 Text) (Data from NEFSC Oceanography Branch) and wind climatology for 2000–2012 determined from nearby NOAA buoys (NDBC GB Station 44011; MA Station 44009), calcium carbonate production as a function of primary production, a primary production climatology estimated from ocean color data from years 2000–2010, and respiration. The primary productivity climatology utilizes the monthly data (grid size 2160 x 4320) from 2003–2012 [47] (http://www.science.oregonstate.edu/ocean.productivity/index.php). Both wind speeds and primary productivity data vary according to a regular seasonal sinusoid, as expected (Table 1) (for winds, MA: r^2^ = 0.97, RMSE = 2.85; GB: r^2^ = 0.94, RMSE = 6.08) (for primary productivity, MA: r^2^ = 0.78, RMSE = 0.12; GB: r^2^ = 0.96, RMSE = 0.10). In the biogeochemical model’s deep box, calcium carbonate production is explicitly modeled, as is organic matter remineralization. The biogeochemical submodel and the scallop submodel run on the same time step (Δt = 1/10 yr) which was chosen to be consistent with sea scallop management models currently used by NOAA NMFS [12,17].

**Table 1:** Parameters from BGC model.

Parameter	MA	GB	unit	Source	Description
*T*_1_*	13.09	9.51	°C	This study; Data from NEFSC Oceanography	T/S model
*T*_2_*	12.00	10.00	°C		
*S*_1_*	32.22	32.47	psu		
*S*_2_*	32.49	32.44	psu		
*A*_1,*T*_	14.48	9.00	N/A		
*A*_1.s_	-1.46	-0.75	N/A		
*A*_2.*T*_	5.21	3.50	N/A		
τ_1,T_	0.14	0.09	yr^-1^		
τ_1.S_	0.11	0.27	yr^-1^		
τ_2_	0.19	0.15	yr^-1^		
*φ*_*1*,*s*_	-0.35	-0.45	yr		
*φ*_*1*.*T*_	-0.63	-0.97	yr		
*φ*_*2*,*T*_	-1.96	-1.54	yr		
*A*_*PP*_	0.27	0.58	gC m^-2^ d^-1^	This study; http://www.science.oregonstste.edu/ocean.productivity/index.php	PP
*φ*_*PP*_	-1.92	-2.11	yr		
*β*_*PP*_	1.07	1.04	gC m^-2^ d^-1^		
*Remin*	1.00	0.80	N/A	GB [48]; MA:[49] Data from NEFSC Oceanography	BGC
*PICPOC*	0.04	0.04	N/A		
*F*_*ratio*_	0.25	0.25	N/A		
*K*_*s*_	10^-5^	10^-5^	m^2^ s^-1^		
*h*_1_	22.00	25.00	m		
*h*_2_	38.00	45.00	m		
*A*_*U*_	22.74	-33.84	m^2^ s^-1^	This study; Data source NDBC	Wind
*φ*_*U*_	4.41	4.14	yr		
*β*_*U*_	52.05	54.23	m^2^s^-2^		

Items have different values for Georges Bank (GB) and Mid Atlantic (MA).

The scallop submodel (details in Eqs. SI11-SI18 in S1 Text and Table 2) draws heavily from the von Bertalanffy growth function- (VBGF-) and growth matrix-based [50] population dynamic models used by NOAA for providing management advice to the sea scallop fishery [Scallop Area Management Simulator (SAMS), and the Catch-at-size-analysis (CASA) [12]]. All growth, weight, and mortality parameters were drawn directly from NOAA NMFS documentation [12]. Unlike the SAMS model, the submodel developed here is not completely spatially explicit. It does, however, separate the two populations of Georges Bank (near 41°N, 69°W) and the Mid-Atlantic Bight (near 39°N, 74°W). The model assumes that recruitment is naturally variable each year using random (Gaussian) stochasticity around the stock-recruit relationship.

**Table 2:** Parameters for the scallop submodel.

Parameter	GB	MA	Unit	Source	Description
*H*_*∞*_	144.9	132.1	mm	[12]	Growth Matrix
*σH*_*∞*_	14.5	13.1	mm	[12]	
*K*_*i*_	0.429	0.527	t^-1^	[12]	
*σK*_*i*_	0.11	0.135	t^-1^	[12]	
*a*_*L*_	23.0	15.5		[12]	Selectivity
*b*_*L*_	0.221	0.139		[12]	
*a*_*mw*_	-10.70	-10.25		[51]	SH/MW relationship
*b*_*mw*_	2.94	2.85		[51]	
*MW*_*∞*_	54.7	46.6	g	[12]	
*M*	0.12	0.15	yr^-1^	[12]	Natural mortality
*I*	0.1	0.2	yr^-1^	[12]	Incidental Mortality
*D*	0.2	0.2	yr^-1^	[12]	Discard Mortality
In(*a*_R_)	20.17	21.35	Millions	[17]	Recruitment
*σlna*_*R*_	0.202	0.341			
In(*γ*_R_)	6.664	10.073	mT	[17]	
*σlnγ*_R_	2.871	0.464			
*ρ*	0.775	0.904			
*F*_*msy*_	0.38	0.38	yr^-1^	[52]	Fishing mortality
*F*_*abc*_	0.32	0.32	yr^-1^	[52]	Fishing mortality

Items, except for discard mortality rates, have different values for Georges Bank (GB) and Mid Atlantic (MA).

The socioeconomic submodel (details in Eqs. SI19-SI30 in S1 Text and Table 3) is based on statistical relationships determined through market and economic analyses from NMFS [12,52,53] combined with static economic theory from Moore [54] and economic decision making tools adapted from Nobre et al. [55]. It uses a Cobb-Douglas production function dependent on biomass and number of days fished [56], along with output from the scallop submodel, to estimate industry-wide catches. This production function (Eq. SI19 in S1 Text) was fit to the annual reported landings (NMFS), reported stock biomass [12], and the days at sea (*DAS*)[52] derived using the vessel trip report (VTR) database [52] from 2000–2009. The production function fit the CASA July 1 biomass and fleetwide *DAS* very well (Table 2, r^2^ = 0.97, p <0.0001), and all coefficients were significant at the 0.99 level. Additionally, autocorrelation and heteroskedasticity were not observed as denoted by a Durbin-Watson test (p = 0.680, DW = 2.61) and a White test (p = 0.343), respectively.

**Table 3:** Parameters and quantities in the socioeconomic submodel.

Parameter	Value	Unit	Source	Description
*e*_*p*_	-0.368	N/A	[54]	Own-price elasticity of demand
*e*_*i*_	1.83	N/A	[54]	Income elasticity of demand
*GRT*	166.2	Ton	[12]	Average tonnage
*α*	1.195	N/A	This study	production function (*BIO*)
*β*	0.449	N/A	This study	production function (*DAS*)
*A*	-9.95	lb^-1^ d^-1^	This study	production function
*Q*	Varies	Lb	[12]	Landings
*C*	S1 Text, Eq. SI27	$	[54]	Total fishery operating costs
*w*	Varies	$ d^-1^	[12]	Cost of fishing per day at sea
*CREW*	7	—	[12]	Average crew per vessel
*FUEL*	Varies	$ gal^-1^	[52]	Cost of fuel
*DFT*	1,0	—	[52]	Indicates dredge or trawl gear
*TRW*	1,0	—	[52]	Indicates dredge or trawl gear
*MC*	Eq 1	$ lb^-1^	This study	Marginal cost
*MR*	Varies	$ lb^-1^	This study/[52]	Marginal revenue
*MCS*	Varies	$ lb^-1^	This study/[52]	Marginal crew share
*P*_*i*,*t*_	Varies	$ lb^-1^	[52]	Price of scallops in market class *i* for year *t*
*MCT*_*i*_	Varies	lb^-1^	[52]	Mean scallop count in market class *i*
*IP*_*t*_	Varies	$ lb^-1^	[52]	Price of imports in year *t*
*R*_*fuel*_	1.1	%	[12], reference case	Rate of increase of diesel fuel prices

At the beginning of each model year (i.e., the socioeconomic model time step is 10Δ*t*), the socioeconomic model determines industry costs, revenues, and profits by maximizing profits and assuring that hard limits are satisfied (Fig 2). Furthermore, the scallop and socioeconomic models are tightly and explicitly linked through the total catch for the year (*Q*, lb yr^-1^), which is determined using data from both the submodels. Scallops caught are removed from each size bin in the scallop submodel to generate landings in the socioeconomic submodel. The number of scallops caught per size class in each time step (Eq. SI16 in S1 Text) is calculated separately for scallop populations in GB and MA, where the total catch (*Q*_*t*_, Eq. SI17 in S1 Text) determined by the socio-economic submodel is separated into catch from GB and MA by assuming the landings extracted from each location are proportional to the exploitable biomass there (the number of full-grown individuals larger than 90mm). Landings are separated into meat count categories used by the fishery: U10 (10 or fewer scallops lb^-1^), 11–20 (11–20 scallops lb^-1^), 21–30 (21–30 scallops lb^-1^) and 31–40 (31–40 scallops lb^-1^). Finally, the end of year biomass and the percent of scallops landed by weight in each category are passed from the scallop submodel to the socioeconomic submodel.

### Socioeconomic decision making in the model

There are two steps in our model’s decision-making process that incorporate elements of the NMFS [12,52,53], Moore [54], and Nobre et al. [55] approaches. The first step is at the management level where rules are developed based on the population model to set the maximum allowable catch. The second step is at the fleet level where maximum allocated days at sea are determined that sustain the maximum allowable catch level, maximize profits, and satisfy an analysis of marginal operating costs, marginal revenues, and national demand.

The management step uses the year-end population distribution to calculate the allowable biological catch (*Q*_*ABC*_) and the maximum allocated days at sea by integrating the scallop submodel (Eq. SI11-SI18 in S1 Text) for a full year and tracking the catch. When determining *Q*_*ABC*_, total fishing mortality is calculated as in Eq. SI14 in S1 Text, but with fishing mortality (*F*_*t*_) equal to fishing mortality associated with allowable biological catch (*F*_*ABC*_, or 0.32 yr^-1^), which is a reduction of the current fishing mortality that achieves the maximum sustainable yield (*F*_*msy*_, 0.38 yr^-1^); this allows for scientific uncertainty. The populations of GB and MA are again tracked separately, and *Q*_*ABC*_ is the sum of the catch for the year from each location. This approach for calculating *Q*_*ABC*_ is an adaptation of the current area-specific method used in CASA and SAMS.

The second step for the decision-making process first determines the economically optimal catch (*Q*_*t*_) at the fleet level, which can equal or be lower than the allowable biological catch *Q*_*ABC*_. Current management limits fishing effort by setting the maximum number of days at sea for the entire fleet (*DAS*_*max*_). *DAS*_*max*_ is derived from NEFSC relationships that determine landings per unit effort, or number of scallops caught per day at sea. Then *Q*_*ABC*_ and *DAS*_*max*_ become management-set upper limits for calculating the optimal catch (*Q*_*t*_) in the socioeconomic submodel.

Next, marginal costs, marginal revenues, and national demand are calculated. Total fishery operating costs (*C*) are:

$$C=w{\left(\frac{Q}{A*BIO^{\alpha }}\right)}^{\frac{1}{\beta }},$$ Eq (1)

assuming static profit maximization as in Moore [54], where *w* (USD/day) is the cost of fishing per day at sea ([53], presented in Eq. SI27 in S1 Text), and β, α, and *A* are coefficients from the production function (Table 3). The marginal operating cost (*MC*) of fishing is the partial derivative of (Eq 1) with respect to *Q*:

$$MC=\;\frac{\partial C}{\partial Q}=\frac{w}{\beta }\left(\frac{1}{A*BIO^{\alpha }}\right){\;}^{\frac{1}{\beta }}Q^{\frac{1-\beta }{\beta }}$$ Eq (2)

where *BIO* is the total biomass (Eq. SI19 in S1 Text). More detail is available in S1 Text. In the current industry practice, the operating costs are felt by the crew, rather than the boat owners (e.g. Eqs. SI22-SI26 in S1 Text), which sets up a socioeconomic tension between scallop crew and scallop boat owners. For example, if the marginal crew income (*MCI*) is not greater than the marginal cost of fishing, crew would expend more effort for less gain. The *MCI* is calculated as:

$$MCI=\;\frac{\partial NCI}{\partial Q}=\left(\bar{P_{i,t}}-0.05\bar{P_{i,t}}\right)0.52-MC,$$ Eq (3)

where *NCI* is net crew income, and $\overset{-}{P_{i,t}}$ is the price of scallops (Eq. SI21 in S1 Text). Future analyses with this model will explore the role of *MCI* as an additional model diagnostic. As in the Nobre et al. [55] MARKET model, scallop fishermen, including owners and crew, are assumed to be price takers; thus the marginal revenue (*MR*) is assumed equal to price $\overset{-}{P_{i,t}}$

$$MR=\;\frac{\partial GR}{\partial Q}=\frac{\partial }{\partial Q}Q_{t}\bar{P_{i,t}}=\bar{P_{i,t}}.$$ Eq (4)

Here, *GR* is gross industry revenue (Eq. SI20 in S1 Text). To ensure that scallop landings are never in excess of US demand, the US national demand (*D*_*t*_) for scallops is estimated initially as the sum of landings minus exports plus imports, but afterwards as:

$$D_{t}=\;D_{t-1}\left(1+r_{d}\right).$$ Eq (5)

where *D*_*t-1*_ is the demand from the previous year and *r*_*d*_ is the rate of change of demand between the previous and current years, calculated as in Nobre et al. [55]:

$$r_{d}=e_{p}r_{p}+e_{i}r_{i}$$ Eq (6)

where *e*_*p*_ is the price elasticity of demand [54], *r*_*p*_ is the rate of change in price from the current and previous years, *e*_*i*_ is the income elasticity of demand [54], and *r*_*i*_ is the rate of change in income (Table 2).

The decision-making component uses *Q*_*ABC*_ and *DAS*_*max*_ and determines the optimal fleetwide *Q*_*t*_ and *DAS* by varying *DAS*. The decision-making component checks annually to ensure that the following conditions are upheld: profits are maximized, *MC ≤ MCI*, *Q*_*t*_ − *export ≤D*_*t*,_ *Q*_t_ ≤*Q*_*ABC*_, and *DAS* ≤ *DAS*_*max*_ (Fig 2). To link the socioeconomic model to the scallop model, the optimal *DAS* fished are related to fishing mortality *F*_*t*_ (yr^-1^):

$$F_{t}=DAS\;F_{das}$$ Eq (7)

where *F*_*das*_ (Eq. SI30 in S1 Text) is fishing mortality from a single day at sea, *DAS* is the total number of days fished fleet wide per year. *F*_*t*_ is used as the initial *F*_*t*_ in Equation SI14 in the scallop submodel (see S1 Text).

### Linking the scallop and biogeochemical submodels

There are several ways the biogeochemical model influences the sea scallop model (Fig 2). The surface box in the biogeochemical model is assumed to influence the larval scallop stage and the deep box is assumed to influence the adult stage. We assume that biogeochemistry can influence stages of the scallop life cycle by multiple routes: 1) ocean acidification on scallop recruitment, 2) deep water temperature effects on scallop growth, and 3) deep water ocean acidification effects on scallop growth.

Scallop recruitment may be directly correlated with calcium carbonate saturation state. Recruitment is the number of individuals who survive the larval and juvenile stages to 40mm shell height[57], and this influences the overall stock that survives to grow into harvestable stock. Based on information from other bivalves [26,28,58] including the very similar king scallop (*Pecten maximus L*.) [30], we assume that calcium carbonate saturation state *Ω* is correlated with sea scallop recruitment. Using the data from Andersen et al. [30] for king scallops, we scale sea scallop larval survival based on the two-year lagged surface *Ω*_*ar*_ (saturation state of aragonite) at the time of spawning by:

$$scale\;factor=\frac{\%\;survival}{45\%}=\frac{\left(20.5\Omega_{ar}-3.7\right)}{average\;survival}.$$ Eq (8)

The average larval survival from a given brood is equal to the control survival from the study (45%) [30]. We simulate impacts on recruitment by reducing the spawning stock biomass by the scale factor determined in (Eq 8), which has the effect of reducing larval production by this scale factor. A Beverton-Holt stock-recruit relationship is then used to determine adult recruitment. Although temperature has been hypothesized to affect recruitment in pectinid species [11,59], no quantitative information is available to build a similar scaling relationship for this species, so we did not alter recruitment by temperature in the model.

The model allows deep-water temperature from the biogeochemical model to influence the growth of recruits after settlement in the scallop model. Elevated temperatures increase growth rates and metabolism to a point, then impede growth beyond a limiting high temperature [11,16]. Because scallops recruit at age 2, we used the 2-year lagged mean temperature and allometric relationships described in Heilmayer et al. [36] to calculate the relative change in the Brody growth coefficient (*K*) with temperature and recalculate the growth matrix **G**_**t**_ for every time step of the scallop model (See Eq. SI12 in S1 Text). This then affects the ‘overall growth performance’ index (OGP = K*M_∞_), where M_∞_ is the mass at size *H*_*∞*_, the location-specific maximum length (Table 2) [60]. The OGP of different species of scallops can be compared across latitudinal and temperature gradients using an Arrhenius model (See Eqs 2, 5, and Fig 2 in [36]). Temperature does not exceed the maximum value tolerated by this species at any time in the modeled period.

**Fig 3:**
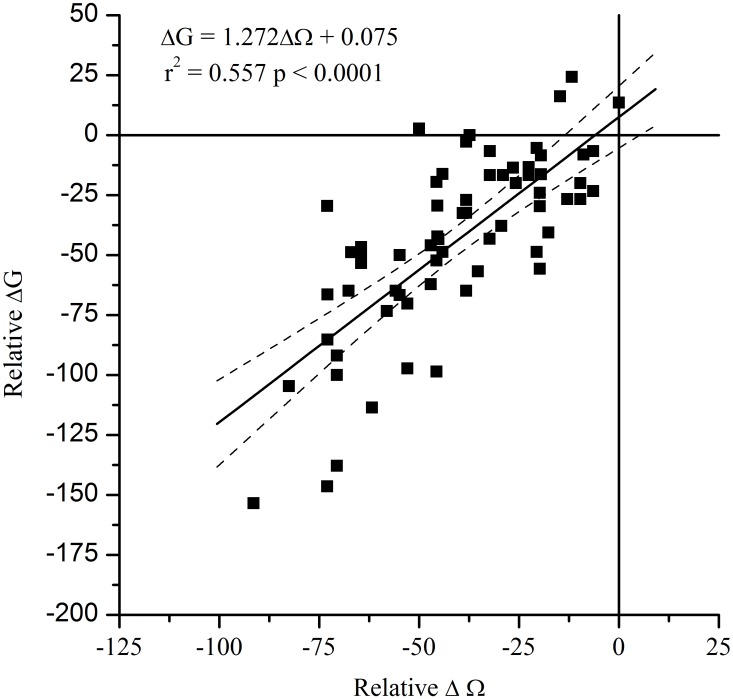
Relative change in adult/juvenile (>40 mm) bivalve growth or calcification vs. relative change in Ω from literature studies. Includes 8 different species from 6 different studies. Dashed lines are 95% confidence intervals.

Via a direct correlation, the model also allows decreases in saturation state to decrease both mollusk growth and calcification rate [61,62]. The effects of ocean acidification on *P*. *magellicanus* have not been studied, so we performed a meta-analysis of literature that reported impacts of ocean acidification on adult mollusk growth or calcification and that was part of the compilation used by Kroeker et al. [62]. We included only bivalves in our analysis, which resulted in 6 studies reporting growth or calcification rates from 8 different species spanning a variety of ecological niches, from intertidal estuarine species to coastal shelf species [27,63–67]. We discarded one study using the Antarctic bivalve *Laternula elliptica* [64] because the slow growth rate of the species resulted in no observed growth from either the control or experimental groups over the timeframe of that study. The resulting data from 7 bivalve species in 5 different studies demonstrated a significant correlation between decreasing saturation state and calcification or growth rate (Fig 3). As these studies were completed using a wide range of saturation states, temperatures, and measured responses, we use the relative change in growth rate or calcification rate from the control compared to the relative change in Ω from the control. For studies that only reported no significant difference, we assume the relative change in growth, ΔG, is 0. The resultant relationship is ΔG = 1.272ΔΩ + 0.075 (Fig 3; r^2^ = 0.56, p <0.0001). To convert between this relationship and the model space, we assume that the Brody growth coefficient *K* (Eq. SI12 in S1 Text) is directly proportional to the growth or calcification rates reported. Any relative change in growth or calcification rate with saturation state (Fig 3) corresponds to a relative change in *K* from the control (the initial measured *K*, Table 1). The change in the growth parameter *K* due to temperature is calculated (Δ*K*_*T*_), then the change due to saturation state is calculated separately (Δ*K*_*Ω*_). To be conservative in our estimates, we assume the changes in growth rate caused by temperature and saturation state occur additively. The value of *K*, adjusted for temperature and *Ω*, used in Eq. SI12 in S1 Text then becomes:

$$K_{T,\Omega }=K\;+\;\Delta K_{T}\;+\;\Delta K_{\Omega }.$$ Eq (9)

Modeled saturation state never drops below the lowest values in observational studies that contributed to this relationship. As saturation state never decreases below *Ω* = 1 during the period investigated, scallop shells would not be expected to dissolve in situ, and this very minor feedback from scallops to ocean biogeochemistry was not included in the model.

### Model initialization and data

The fully coupled IAM was tested by initializing with data from year 2000, and model results were compared to actual landings, revenue, biomass, number, and scallop size distribution from NEFSC for the period 2000–2012 (2009 for biomass and number) and available biogeochemical data from the NEFSC Oceanography Branch (temperature and salinity), the gridded SOCAT database (pCO_2_), and the GOMECC I and GOMECC II cruises (SOCAT:[68], GOMECC I: [24], http://www.aoml.noaa.gov/ocd/gcc/GOMECC1/, GOMECC II: http://www.aoml.noaa.gov/ocd/gcc/GOMECC2/, Wanninkhof pers. comm.). Initial conditions for the BGC model are given in Table 4. The scallop model was initialized with scallop size distribution from the NMFS sea scallop surveys and number of individuals >40mm estimated for July 1, 2000 from the NMFS CASA model. Although the stock-recruit relationships represent the long-term mean in recruitment, there is considerable interannual variability in recruitment in scallop populations. Because interannual variability in recruitment is such a strong driver of sea scallop biomass and abundance, in order to test the model behavior, annual recruitment numbers from the CASA model were used from 2000–2012. The hindcasts also used the reported 2000–2012 per capita disposable income (U.S. Bureau of Economic Analysis, data accessed 10/1/2013), corrected to 2011 dollars using the CPI (Bureau of Labor Statistics, data accessed on 1/16/2014), mean annual import prices (NMFS Foreign Trade, accessed 7/10/2013 corrected to 2011 dollars), and annual scallop exports (NMFS Foreign Trade, accessed 7/10/2013) for the price model. Initial conditions for the scallop and socioeconomic submodels are given in Table 5.

**Table 4:** Initial conditions for the biogeochemical submodel.

Parameter	GB	MA	Unit	Source	Description
*T*_1_	23.29	16.67	°C	NEFSC Oceanography	Surface temperature
*T*_2_	8.52	9.06	°C		Deep temperature
*S1*	31.67	32.19	ppt		Surface salinity
*S*_*2*_	32.95	32.67	ppt		Deep salinity
*DIC*_*s*_	1890.1	1987.6	μmol KgSW^-1^	GOMECCII, R. Wanninkhof, pers. Comm.	Surface DIC
*DIC*_*d*_	2019.0	2042.8	μmol KgSW^-1^		Deep DIC
*TA*_*s*_	2160.6	2206.6	μmol KgSW^-1^		Surface TA
*TA*_*d*_	2125.7	2212.4	μmol KgSW^-1^		Deep TA

Items have different values for Georges Bank (GB) and the Mid Atlantic (MA).

**Table 5:** Initial conditions for the socioeconomic and scallop submodels for year 2000.

Parameter	Value	Unit	Source	Description
*FC*_0_	191167	$	[52]	Vessel fixed costs (2001–2007 mean)
*ASSN*_0_	1610	$	[52]	Association fees per vessel
*COMM*_0_	3446	$	[52]	Communication fees per vessel
*BIO*_0_	172458000	Lb	[12]	Biomass
*DAS*_0_	25849	days	[52]	DAS
*FUEL*_0_	1.56	$	[52]	Fuel price
*w*_0_	1184	$/day	[52]	Cost/DAS
*PCDI*_0_	25946	$	[69]	Per capita disposable income
*D*_0_	78903000	$	[70,71]	Demand = landings-exports+imports
*R*_0_	3728	millions	D Hart, pers.comm.	CASA recruitment
*P*_*U*10_	8.94	$/lb	[52]	U10 price
*P*_1020_	6.73	$/lb	[52]	10–20 price
*P*_2030_	6.02	$/lb	[52]	20–30 price
*P*_3040_	6.08	$/lb	[52]	30–40 price
LAND	32161800	Lb	[70]	Landings
*N*_*MA*_	3523	millions	[12]	CASA number in MA
*N*_*GB*_	3129	millions	[12]	CASA number in GB
*Export*	7224800	Lbs	[71]	Exports all scallop
*IP*0	3.98	$	[71]	Import price
*Import*	53966000	lbs (all)	[71]	Imports all scallop
*PCTLAND*_*U*10_	7	pct	[52]	Percent landed U10
*PCTLAND*_1020_	20	pct	[52]	Percent landed 11–20
*PCTLAND*_2030_	42	pct	[52]	Percent landed 21–30
*PCTLAND*_*3040*_	21	pct	[52]	Percent landed 31–40

All dollars reported in 2011 USD.

### Model sensitivity testing

The model was tested for sensitivity to model parameters by using a Monte Carlo approach [72]. Distributions were assigned to 38 of the model parameters based on either literature values or values were assumed to be multivariate normal from our least squares regressions of the climatologies (Table 1). The model was run for 500 iterations, drawing the parameters from their various distributions. The model was run from 2000–2012 and forced with scallop recruitment values estimated from NMFS survey assessments. Stepwise multiple linear regression was performed by fitting model parameters to the 2012 biomass, landings, and revenues to identify parameters that had the most impact on these model results, which are key indicators describing the social-ecological system (S1 Table).

## Results

### Scallop model skill

**Fig 4:**
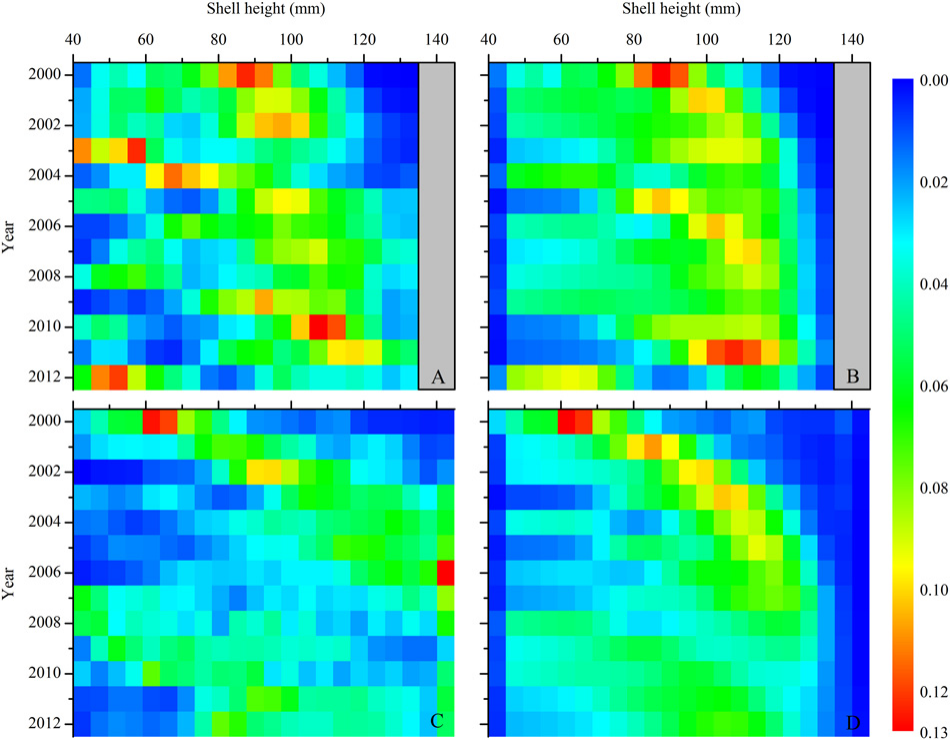
A) Actual and (B) modeled relative scallop shell height distribution from Georges Bank based on number of individuals in each 5mm size class. Data from D. Hart, from NMFS scallop surveys. Data from year 2000 are model initial conditions.

The scallop model (Fig 2, in blue) correctly reproduced the trends in shell height distribution from both locations (Fig 4). During all years, the model scallop size distribution was not significantly different from the whole-stock size distribution reported from the NMFS sea scallop surveys using 2-sample Kolmogorov-Smirnov tests (MA: p >0.05, KSstat <0.369; GB: p >0.05, KSstat <0.312, n = 22 for all years).

**Fig 5:**
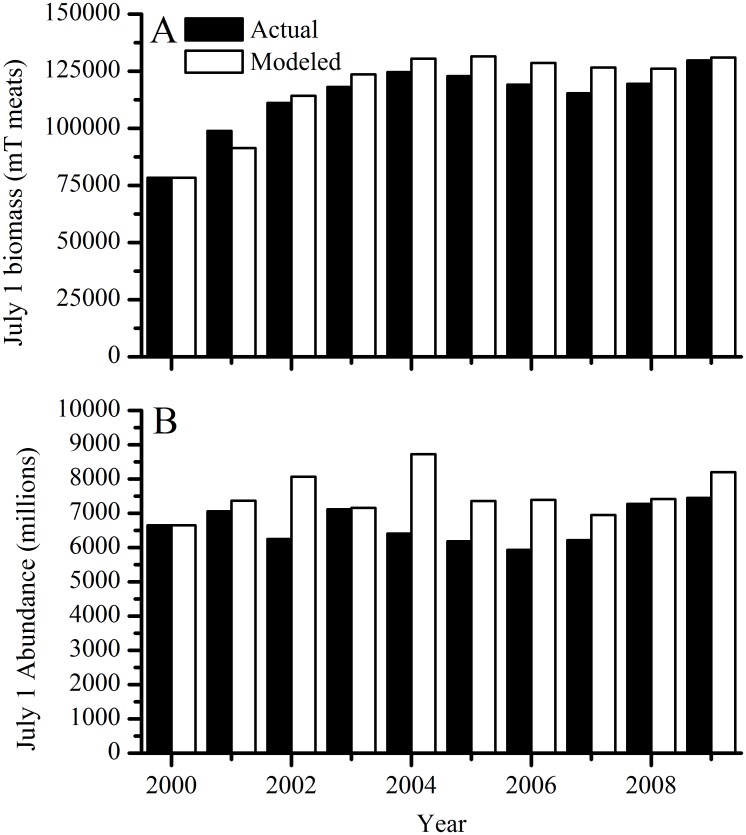
Actual (NEFSC 2010, CASA estimated July 1) and modeled whole stock (A) biomass and (B) abundance. Data from year 2000 are model initial conditions.

Whole stock modeled biomass (Fig 5A) and abundance (Fig 5B) agreed with CASA modeled whole stock July 1 biomass and abundance from 2000–2009 [12]. Mean CASA July 1 scallop abundance was 6654 million individuals (range 5933–7446) and scallop biomass was 113790 metric tons (mT) meats (range 78390–129703). Mean modeled abundance was 7528 million individuals (range 6652–8724) and biomass was 118200 mT meats (range 78390–131450). The average absolute model-data error for abundance and biomass was 15.3 ± 12.6% and 3.97 ± 5.03% (SD, n = 10), respectively.

**Fig 6:**
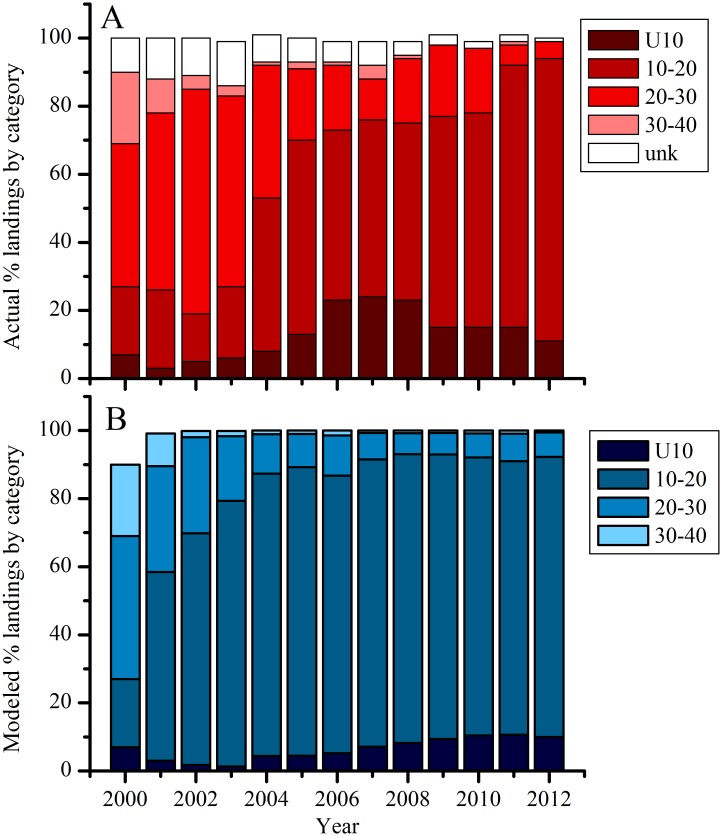
(A) Actual data from NMFS (may not add up to 100%) and (B) modeled scallop landings by category. Category represents range of meat count/lb. Actual data reported in [52](may not add up to 100). Note actual 2012 from months 1–3. Data from year 2000 are model initial conditions.

Scallop landings by size class determined from the population model also agreed well with trends in landings from 2000–2012 (Fig 6) and accounted for changes in gear selectivity, which occurred several times throughout the period, that select for larger sized scallops. Mean reported and modeled U10 scallop landings from this time period were 12.9% (range 3–24%) and 7.9% (range 1.2–11.6%), respectively. Mean 11–20 reported and modeled landings were 47.6% (range 14–77%) and 74.4% (range 20.0–85.7%), respectively. Mean 21–30 reported and modeled landings were 29.0% (range 6–66%) and 13.8% (range 5.2–42.0%), respectively. Mean 31–40 reported and modeled landings were 3.7% (range 0–21%) and 3.2% (range 0.5–21%), respectively [52]).

### Economic model skill

**Fig 7:**
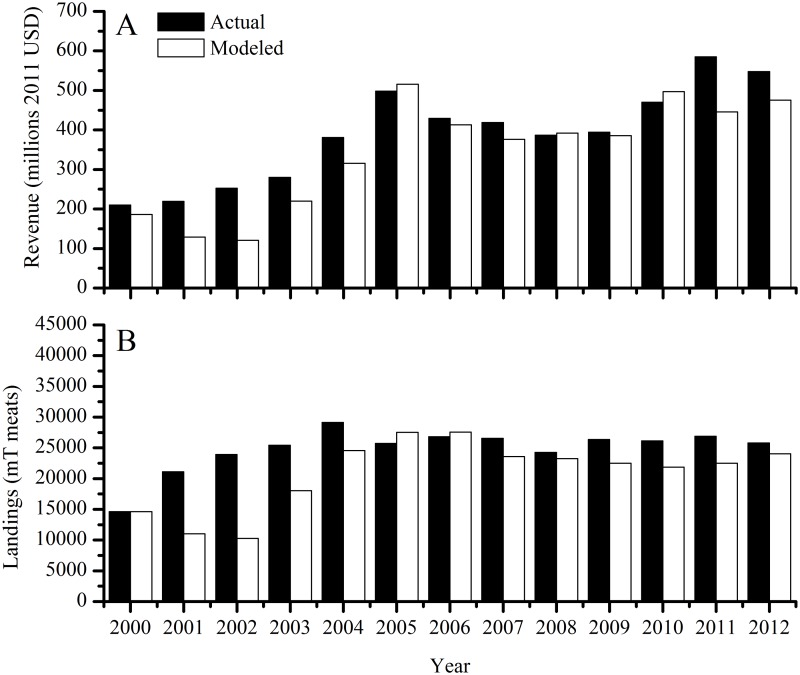
Actual (NMFS commercial landings accessed Jan. 28, 2014) and modeled ex-vessel (A) revenue and (B) landings. Data from year 2000 are model initial conditions.

Scallop ex-vessel prices matched well the reported prices in both trend and magnitude (not shown) resulting in revenues that agree well with the reported revenues (Fig 7A) and landings (Fig 7B) from 2000–2012. Mean annual reported and modeled revenue for this period was 349 million USD (range 157–573 million USD) and 343 million USD (range 149–483 million USD), respectively. Mean annual reported and modeled landings were 24835 mT meats (range 14619–29140 mT meats) and 20535 mT meats (range 12713–25528 mT meats), respectively. Average absolute model-data error in revenues and landings was 13.2 ± 11.5% and 19.1 ± 17.2%, respectively (SD, n = 13).

### Biogeochemical model skill

**Fig 8:**
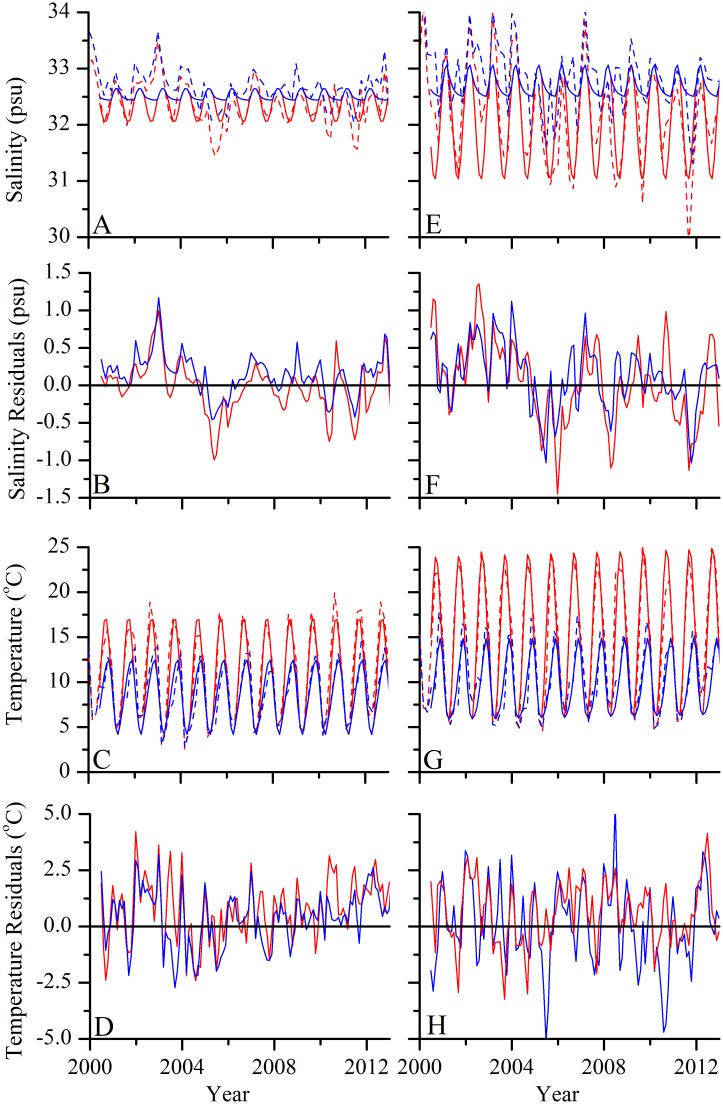
Salinity, salinity residuals, temperature, and temperature residuals from the two box models (solid) calibrated to data collected during bimonthly oceanographic cruises (dashed) within a 1° x 1° area representative of conditions on (A—D) Georges Bank and (E—H) in the Mid-Atlantic. Surface conditions are given in red and bottom conditions are given in blue. Data from NEFSC Oceanography branch.

The simplified two box model reproduced seasonal cycles of temperature, salinity, and annual water column stratification and mixing well (Fig 8). Model RMSE for the surface and deep temperature was 1.58 and 1.31°C, respectively and RMSE for the surface and deep salinity was 0.39 and 0.37 psu, respectively.

**Fig 9:**
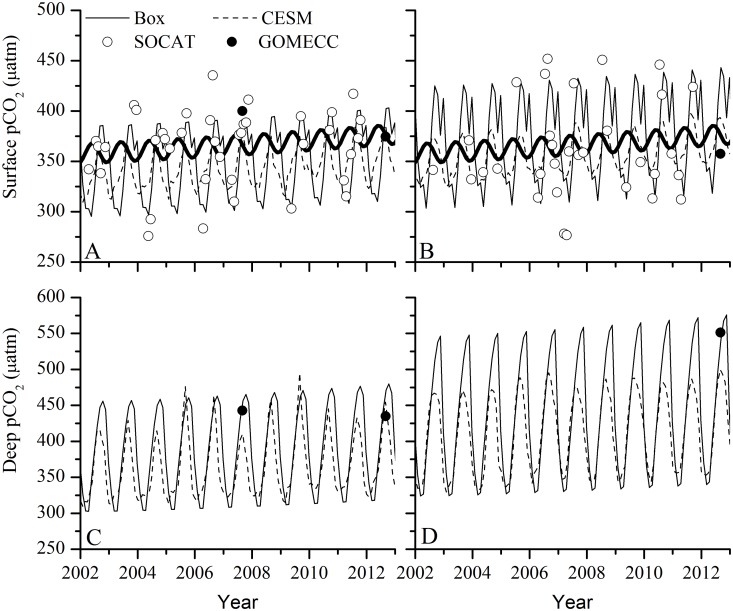
(A) Georges Bank and (B) Mid-Atlantic surface pCO_2_ from the box model (solid), the global CESM model (dashed), and atmospheric CO_2_ forcing (heavy solid). Open circles are from the SOCAT gridded database [68] and closed circles are from the GOMECC I [24] and II (Wanninkhof pers. comm) cruises in 2007 and 2012. (C) Georges Bank and (D) Mid-Atlantic deep pCO_2_ from the box model (solid) and the global CESM model (dashed)[74].

**Fig 10:**
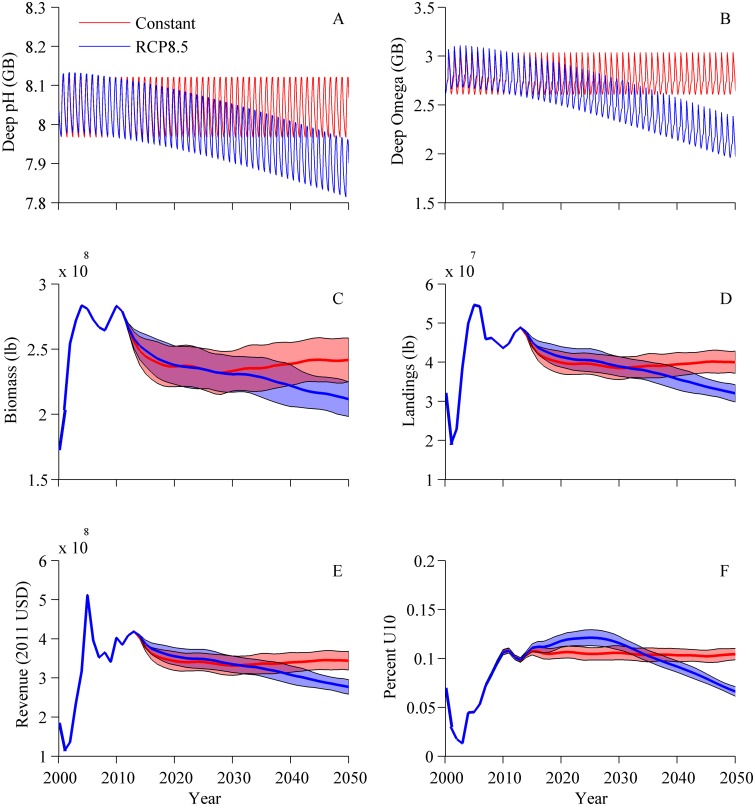
Mean ± SD (n = 100) model forecasts out to 2050 using CO_2_ forcing from RCP 8.5 and 1.4°C SST warming (blue) and forecasts with constant 2008 CO_2_ concentration and temperature (red). Deep box (A) pH and (B) calcite saturation state from Georges Bank, (C) landings, (D) total spawning stock biomass (scallops >40mm shell height), (E) revenue (2011 USD), and (F) fractional landings in category U10 (meat weight 10 and under scallops/lb).

The magnitude and timing of seasonal cycles of DIC and TA agree well with model estimates from the global Community Earth System Model (not shown), producing pCO_2_ values that agree very well between the two models in both the surface and deep boxes (Fig 9), as well as with the gridded SOCAT surface pCO_2_ data available from the representative model region (Fig 9A)[73] and the profiles from the GOMECC I and II cruises (Fig 10). Modeled pCO_2_ values are consistently somewhat (~30 ppm) lower than the ranges reported by Rebuck and Hare [23]. Normalized RMSE of the model and SOCAT pCO_2_ database for GB was 7.0% and for MA was 13.2%. Model-data residuals from both GB and MA do not have observable patterns across the time series and are normally distributed (KS test, p = 0.459, KS stat = 0.133, n = 39 for GB; p = 0.759, KS stat = 0.114, n = 32 for MA).

### Model sensitivity

Of the 500 Monte Carlo simulations, 67 did not converge because the gear selectivity fished out the largest scallops quickly. The remaining 433 simulations showed wide variability in 2012 biomass (S2 Fig), landings and revenue, ranging from 30400–265000 mT, 1910–42500 mT, and 4.0–79 million 2011 USD, respectively. Stepwise MLR identified 10 total parameters that were significantly driving the model results explaining 79.3, 86.3, and 78.9% of the variability in biomass, landings, and revenue, respectively (S2 Table).

### Fully integrated scenarios

Model runs varied beyond 2012 owing to stochastic recruitment (Fig 10). Under the RCP8.5 scenarios until 2050, projected mean annual surface box pH decreases from 8.05 to 7.91 in MA and from 8.08 to 7.94 in GB, respectively. Projected deep box pH decreases from 8.05 to 7.85 in MA and from 8.05 to 7.89 in GB, respectively. Projected Ω (calcite) in the surface box decreases from 3.35 to 2.65 in MA and from 3.23 to 2.51 in GB, respectively. Projected Ω (calcite) in the deep box decreases from 2.64 to 1.99 in MA and from 2.80 to 2.11 in GB, respectively (shown for GB in Fig 10A and 10B). Under the constant CO_2_ scenario, projected total landings (Fig 10C), biomass (Fig 10D), revenues (Fig 10E), and distribution of landings by size class (e.g. Fig 10F, shown for U10) stabilize after 2020, but decrease under the RCP8.5 scenario. Compared to the constant CO_2_ scenario, under the climate change scenario RCP8.5, the percent of landings from the largest category of scallops (U10) decreased from a mean ± SD of 11.0 ± 0.3% in 2012 to 6.6 ± 0.5% in 2050 (Fig 10A).

## Discussion

The development of this sea scallop IAM offers new possibilities for considering anthropogenically driven global change in fisheries harvest planning. Because our primary goal is to enrich resource management deliberations by offering both short- and long-term insight into the system rather than to replace current fisheries management models, our simplified, modular approach to building this IAM is appropriate. It can be easily updated as new information becomes available, and complexity can be built into the submodels as research questions demand.

When run under “control” conditions, or present-day CO_2_ and harvest levels held constant through time, the model demonstrates stable behavior and good skill in reproducing the past ten years of harvests. Model deviations from the observed parameters are explained either by spatially explicit details we did not include in the model (e.g., rotational management and closed areas) or by natural processes (e.g. a massive die-off of large scallops between 2004 and 2004 in MA populations [75]) that occurred during some years. For example, the model’s underestimation of the relative number of scallops in the largest size class in both MA and GB (Fig 4) is because rotational management practices and permanently closed areas that NMFS uses to maximize scallop biomass and yield are not included in the model. This management scheme allows scallops to grow very large in areas closed to fishing and thus attain sizes that are at or larger than the modeled H_∞_, while in the model, scallops are removed as they enter the larger size bins. The model overestimates biomass and abundance in 2004 compared to observations (Fig 5) because a large die-off of small scallops between 2003 and 2004 occurred in MA populations [75], which was not incorporated into the model. This causes the model to overestimate abundance for several years afterwards as the extra scallops grow and are harvested. Finally, the large difference in landings and revenues in the early 2000s (Fig 7) is due to changes in selectivity imposed in the model to reflect NMFS altered selectivity periods (2001–2003, 2004–2012 in GB, see NEFSC 2010 Figure B-26). During the 2001–2003 period, NMFS uses a domed selectivity for GB to simulate a large biomass of scallops that were located in closed areas, and thus unavailable for harvest [12,17]. Using domed selectivity is one method for including spatial variability such as marine protected areas into fisheries population models [76] but it does not provide the best possible model-data fit for this species [17]. We expect that these deviations would decrease if our model included more spatially explicit details.

The simple biogeochemical model containing the major oceanographic features relevant in the GB and MA regions, temperature and salinity-driven mixing and air-sea CO_2_ uptake, reproduced observations of carbonate chemistry well in these regions. The good agreement between modeled and surface pCO_2_ from the SOCAT database (NRMSE 13.2% for MA and 7.6% for GB) from both regions suggests that the model parameterizations are capturing the major processes that drive the local carbonate chemistry. Furthermore, the modeled deep carbonate chemistry agreed well with data from the two GOMECC cruises, although more observations would be necessary to evaluate this component of the model in detail. The more complex oceanographic features of each region, such as horizontal flow, were omitted for simplicity, and leaving them out did not noticeably decrease the skill of the biogeochemical submodel.

A central challenge of building this model is that no studies of ocean acidification’s effects have been completed on *P*. *magellanicus*. To close this data gap, we parameterized growth rate and saturation state for post-settlement adults using a relationship based on other adult bivalve responses, but this has limitations. Despite the availability of studies correlating saturation state and physiological processes, no mechanistic relationship has been proposed to date that links these variables; saturation state is only a proxy for what is likely to be several linked causal mechanisms. The studies used to develop this statistical relationship span a wide range of pH values which are more extreme than those likely to influence scallops by 2100, raising the question of whether this relationship is driven by these extremes. For example, the control values ranged from 8.15–7.99, while the experimental values ranged from 8.09–7.00, depending on source water and pCO_2_ experimental treatment. Restricting our analysis to only values that might be expected by 2100 (pH declines of <0.3), this relationship is still significant and fairly strong for saturation state (p <0.0001, *n* = 49, *F*_*n*_ = 54.45, r^2^ = 0.29). If we include studies on bivalves identified as juveniles into the analysis, this adds an additional 8 studies and 5 species [32,58,77–82] bringing the total to 13 separate studies with 12 different species. Although this would increase the scatter in our relationship, a significant decline in relative growth still occurs with saturation state (see S2 Fig), further supporting our approach. Although we are confident that this relationship is appropriate for the purposes of this study, intraspecific variability in adult responses has been noted [83]. Another unanswered question is whether the timing of early larval CO_2_ exposure that affects later development in other species [84,85] might also occur in *P*. *magellanicus*.

Ideally, directly measured relationships between growth rate, survival, saturation state and temperature would exist for larvae, juveniles, and adults. But these studies have not been completed for *P*. *magellanicus* at this time. Our assumption that the relationships between life processes and saturation state or temperature are additive is a simplification. Synergistic effects of multiple stressors like acidification and temperature have been observed (e.g., [86–89]) and changes in organism performance noted at higher temperatures [34] can alter organisms’ energy budgets and govern their responses to environmental change. Although our modeled temperatures do not exceed the maximum thermal tolerance of *P*. *magellanicus*, it is likely that at the southern limit, or in inshore habitats, temperatures may exceed this threshold under future warming scenarios. If this is the case, our assumption that temperature will only increase growth may be overestimating scallop production in future projections. However, we believe our approach is appropriate given the very limited experimental data available for this species, and that spatially explicit physical modeling of the US East Coast shelf is beyond the scope of this model.

Another assumption in our model is the treatment of scallop recruitment. Because of the lack of species-specific information on pre-recruits [40,90], we chose to impact scallop recruitment only through the stock-recruit relationship rather than developing a specific pre-recruit model as Punt et al. did for king crab [40]. A reduction in scallop recruitment due to ocean acidification could be a result of many processes that occur between spawning and scallop recruitment, such as decreases in gamete production, fertilization, larval survival, or growth and mortality of juveniles. Depending on the shape of the stock-recruit relationship, this may not affect the fishery in intuitive ways. In Georges Bank, the stock-recruit relationship is flat, and reductions in larval survival would only slightly influence recruitment because it is not thought to be limited by larval supply [12,16]. This may be partly due to the nature of local currents which retain larvae within the gyre over Georges Bank [91]. Rather, recruitment may be limited by other factors such as predation, food availability or suitable habitat for settlement. In areas where the stock-recruit relationship is more linear, this is an indication that recruitment may be limited by larval supply [12,16], and reductions in larval survival will influence recruitment substantially. For example, using specified model parameters for both GB and MA for the stock recruit relationships and modeled stock, if larval survival is reduced by 50%, in the Mid-Atlantic, recruitment would be reduced by 24%, while on Georges Bank, recruitment would only be reduced by 1.6%. The major assumptions required to build this model highlight the strong need for studies of the effects of ocean acidification and warming on *P*. *magellanicus* given its large economic importance and relatively unique life history characteristics (e.g., oceanic, mobile adults) compared to other bivalve species harvested by commercial fisheries in the U.S.

The model indicates that with business-as-usual CO_2_ emissions (RCP 8.5), the assumed additive effects of warming and acidification, and the present harvest rules, sea scallop populations and harvests could decline over time. By 2050, average landings of large scallops and total overall landings could decrease (Fig 10). The antagonistic interaction of warming increasing growth rates, ocean acidification slowing growth rates, and interannual variability in recruitment masks any negative influence of ocean acidification in the short term. Interestingly, increased growth rates from warming outweigh decreasing growth rates from ocean acidification until 2030 when the negative influence of ocean acidification overtakes warming; this can be seen in the larger contribution of U10 scallops to the total landings from approximately 2015–2035 compared to the constant CO_2_ scenario (Fig 10F). Beyond 2030, in an ocean undergoing acidification plus further warming, whose net effect shifts to slow overall growth rates, fewer scallops attain U10 size before they are harvested if current fishing levels are maintained. This is because present scallop harvest minimum sizes and allowable catch limits are optimized for a population that is not experiencing progressive externally imposed environmental pressure. As ocean acidification advances, growth slows even farther, and present-day harvest rules (with fixed *F*_*msy*_, *F*_*ABC*_ and fishery selectivity) become increasingly out of step with the resulting population distribution. This highlights the need to understand ocean acidification’s effect on *P*. *magellanicus* and the importance of managing fishery populations in full acknowledgment of the reality that management will occur against a backdrop of progressive change, rather than constancy. As U10 size scallops bring a higher price than other sizes, this result is quite interesting from an economic perspective, and bears further investigation in future analyses using this model.

## Conclusions

The IAM developed in this study represents another step towards regular inclusion of anthropogenically driven global change into fisheries harvest planning. This IAM for the U.S. *P*. *magellanicus* fishery rigorously incorporates the known, published mechanisms that govern scallop growth, relationships between scallops and their environment, and market dynamics. It is a tool that can be used to explore different combinations of catch limits, temperature, acidification, and even different scallop life-history responses to acidification. Model sensitivity testing indicates that the key life history and fishery parameters (natural and incidental mortality, shell height-meat weight relationships, and selectivity) will be critical to understanding the impacts of ocean acidification on the social-ecological system. Importantly, the model can also be revised as knowledge develops concerning the response of sea scallops to ocean acidification, because several possible impact pathways (on scallop recruitment, growth, and mortality) are explicitly parameterized. Initial runs of the model, assuming continued present-day fishery mortality rates, business-as-usual CO_2_ emissions, a linear negative relationship between ocean acidification and sea scallop growth, and additive interactions of warming and ocean acidification indicate an overall decline in scallop harvests, an initial increase followed by a decline in U10 (largest market size class) scallops, and an accompanying decrease in revenue. Future work will explore different economic and management scenarios and test how potential impacts of ocean acidification on other scallop biological parameters may influence the social-ecological system. In this way, the model complements the population dynamic models used now for fisheries management by providing a window into the broader social-ecological system and the long-term context in which the sea scallop fishery operates. This information will allow managers to make informed decisions that plan for both short- and long-term processes that affect the U.S. sea scallop fishery, a perspective that will also be useful in other high-value, single species fisheries elsewhere.

## Supporting Information

S1 Fig Relative change in adult (>40 mm) and juvenile bivalve growth or calcification vs. relative change in (A) pH or (B) Ω from literature studies. Includes 14 different species from 10 different studies. Blue lines are trends using adult only (Fig 3) and red lines are trends using both adult and juvenile studies. Dashed lines are 95% confidence intervals.
(TIF)

S2 Fig Biomass from Monte Carlo model runs. Thick line is the mean value over 433 model runs, shaded area is ± SD, dashed lines are maximum and minimum values for the model runs.
(TIF)

S1 Table Model sensitivity analysis parameters and distributions. (DOCX)

S2 Table Stepwise multiple linear regression of model parameters to 2012 biomass and landings. (DOCX)

S1 Text Equations for the oceanographic, population dynamic, and socioeconomic submodels. (DOCX)
