# Supplementary figures and images for: An Integrated Assessment Model for Helping the United States Sea Scallop (Placopecten magellanicus) Fishery Plan Ahead for Ocean Acidification and Warming
Source: PLoS One. 2015 May 6;10(5):e0124145. doi: 10.1371/journal.pone.0124145 (PMC4422659; doi:10.1371/journal.pone.0124145)

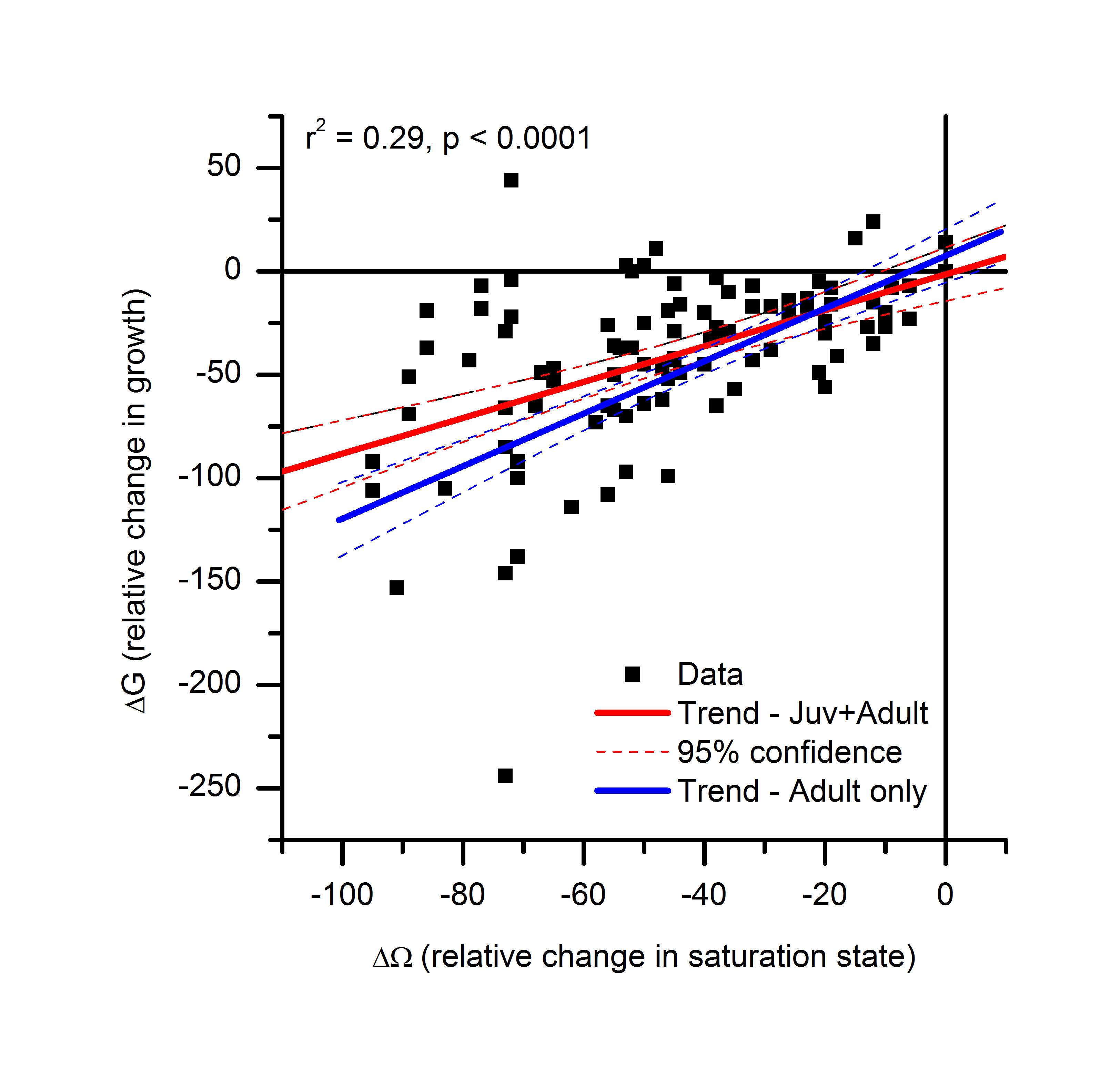

Supplement: S1 Fig — Includes 14 different species from 10 different studies. Blue lines are trends using adult only (Fig 3) and red lines are trends using both adult and juvenile studies. Dashed lines are 95% confidence intervals. (TIF) [file pone.0124145.s001.tif]

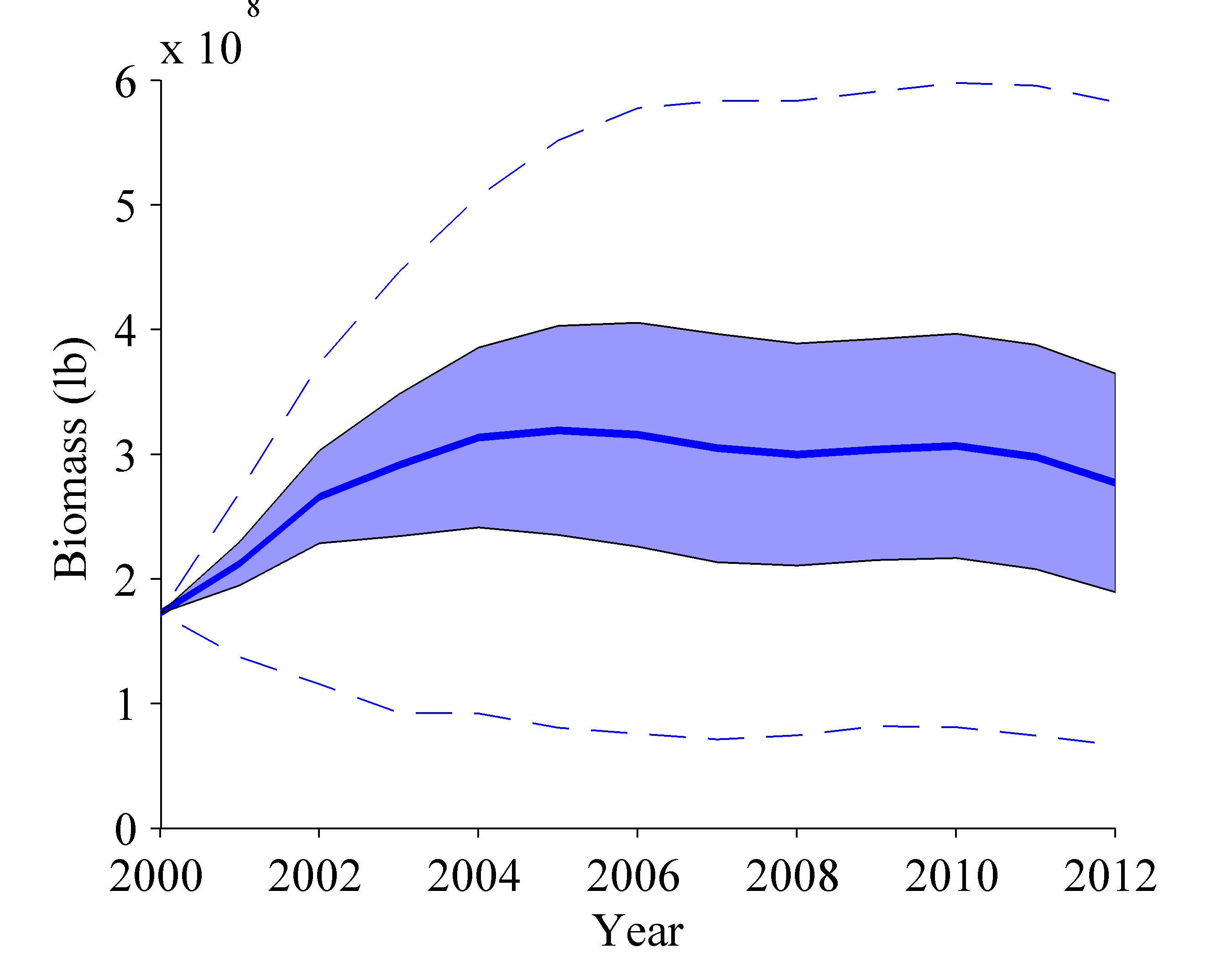

Supplement: S2 Fig — Thick line is the mean value over 433 model runs, shaded area is ± SD, dashed lines are maximum and minimum values for the model runs. (TIF) [file pone.0124145.s002.tif]
