# Supplementary material for: An Integrated Assessment Model for Helping the United States Sea Scallop (Placopecten magellanicus) Fishery Plan Ahead for Ocean Acidification and Warming
Source: PLoS One. 2015 May 6;10(5):e0124145. doi: 10.1371/journal.pone.0124145 (PMC4422659; doi:10.1371/journal.pone.0124145)
Supplement: S1 Table — (DOCX) [file pone.0124145.s003.docx]

**Supporting Information, Table S1**

**An integrated assessment model for helping the United States sea scallop (*P. magellanicus*) fishery plan ahead for ocean acidification and warming**

Sarah R. Cooley, Jennie E. Rheuban, Deborah R. Hart, Victoria Luu, David M. Glover, Jonathan A. Hare, Scott C. Doney

Table S1. Model sensitivity analysis parameters and distributions.

| **Submodel** | **Model Parameters** | **Distribution** | **Source** |
| --- | --- | --- | --- |
| Scallop | Shell height-Meat Weight | Multivariate Normal | [1] |
|  | Selectivity | Multivariate Normal | [1] |
|  | Incidental Mortality | Gamma | [1] |
|  | Discard Mortality | Gamma | [1] |
|  | Natural Mortality | Gamma | [1] |
|  | ∆G - ∆Ω relationship | Multivariate Normal | This study |
| Socio-Econ | Production Function | Multivariate Normal | This study |
|  | Price elasticity of demand | Normal | [2] |
|  | Income elasticity of demand | Normal | [2] |
| BGC | Pic-Poc ratio | Uniform | This study |
|  | Ks | Uniform | This study |
|  | Primary Productivity | Multivariate Normal | This study |
|  | Wind | Multivariate Normal | This study |

1. Hart DR. Quantifying the tradeoff between precaution and yield in fishery reference points. Ices J Mar Sci. 2013;70: 591–603. doi:10.1093/icesjms/fss204

2. Moore CC. Welfare Impacts of Ocean Acidification: An Integrated Assessment Model of the US Mollusk Fishery. National Center for Environmental Economics: U.S. Environmental Protection Agency; 2011 Dec. Report No.: 11-06.
