# Supplementary material for: An Integrated Assessment Model for Helping the United States Sea Scallop (Placopecten magellanicus) Fishery Plan Ahead for Ocean Acidification and Warming
Source: PLoS One. 2015 May 6;10(5):e0124145. doi: 10.1371/journal.pone.0124145 (PMC4422659; doi:10.1371/journal.pone.0124145)
Supplement: S2 Table — (DOCX) [file pone.0124145.s004.docx]

**Supporting Information, Table 2**

**An integrated assessment model for helping the United States sea scallop (*P. magellanicus*) fishery plan ahead for ocean acidification and warming**

Sarah R. Cooley, Jennie E. Rheuban, Deborah R. Hart, Victoria Luu, David M. Glover, Jonathan A. Hare, Scott C. Doney

Table S2. Stepwise multiple linear regression of model parameters to 2012 biomass and landings. Stepwise MLR of 2012 revenues found the same significant parameters as for landings.

| **Model** | **Parameter** | **Unit** | **r^2^** | **p** | **description** |
| --- | --- | --- | --- | --- | --- |
| Biomass | M | Yr^-1^ | 0.502 | < 0.0001 | Average natural mortality |
|  | α_s_ GB | -- | 0.601 | < 0.0001 | GB selectivity 1 |
|  | I MA | Yr^-1^ | 0.674 | < 0.0001 | Incidental mortality MA |
|  | β_s_ MA | -- | 0.726 | < 0.0001 | Meat Weight 1 MA |
|  | I GB | Yr^-1^ | 0.758 | < 0.0001 | Incidental mortality GB |
|  | b_MW_ MA | -- | 0.787 | < 0.0001 | Meat Weight 2 MA |
|  | β_s_ GB | -- | 0.791 | 0.007 | Wind amplitude MA |
|  | A_U_ MA | m^2^s^-2^ | 0.793 | 0.017 | PP offset GB |
| Landings | M | Yr^-1^ | 0.675 | < 0.0001 | Average natural mortality |
|  | I MA | Yr^-1^ | 0.755 | < 0.0001 | Incidental mortality MA |
|  | I GB | Yr^-1^ | 0.796 | < 0.0001 | Incidental moratlity GB |
|  | b_MW_ MA | -- | 0.835 | < 0.0001 | Meat weight 2 MA |
|  | β_s_ MA | -- | 0.855 | < 0.0001 | Selectivity 2 MA |
|  | K-Ω slope | -- | 0.859 | 0.0013 | Growth-Omega slope |
|  | α_s_ GB | -- | 0.861 | 0.0055 | Selectivity 1 GB |
|  | Picpoc GB | -- | 0.863 | 0.028 | PIC to POC ratio GB |
