# Supplementary material for: An Integrated Assessment Model for Helping the United States Sea Scallop (Placopecten magellanicus) Fishery Plan Ahead for Ocean Acidification and Warming
Source: PLoS One. 2015 May 6;10(5):e0124145. doi: 10.1371/journal.pone.0124145 (PMC4422659; doi:10.1371/journal.pone.0124145)
Supplement: S1 Text — (DOCX) [file pone.0124145.s005.docx]

**Supporting Information**

**An integrated assessment model for helping the United States sea scallop (*P. magellanicus*) fishery plan ahead for ocean acidification and warming**

Sarah R. Cooley, Jennie E. Rheuban, Deborah R. Hart, Victoria Luu, David M. Glover, Jonathan A. Hare, Scott C. Doney

*Biogeochemical model*

We adapted a model for seasonal, open-ocean convection to the shelf where the surface box represents the average summer mixed layer and the deep box is the bottom layer beneath the summer thermocline. The model first calculates temperature and salinity for the surface and deep boxes as explained in Rahmstorf [1] assuming an external restoring condition for the following system of equations:

$\frac{{dT}_{1}}{dt}= \frac{1}{\tau_{1,T}}\left( {T_{1}}^{*}-A_{1,T}\cos\left( 2\pi t+\varphi_{1,T} \right)-T_{1} \right)$ Eq. (SI1)

$\frac{{dT}_{2}}{dt}= \frac{1}{\tau_{2}}\left( {T_{2}}^{*}-A_{2,T}\cos\left( 2\pi t+\varphi_{2,T} \right)-T_{2} \right)$ Eq. (SI2)

$\frac{{dS}_{1}}{dt}= \frac{1}{\tau_{1,S}}\left( {S_{1}}^{*}-A_{1,S}\cos\left( 2\pi t+\varphi_{1,S} \right)-S_{1} \right)$ Eq. (SI3)

$\frac{{dS}_{2}}{dt}= \frac{1}{\tau_{2}}\left( {S_{2}}^{*}-S_{2} \right)$ Eq. (SI4)

where the subscripts 1 and 2 denote the surface and deep box, respectively; $T$ and $S$ are temperature and salinity, respectively; $T^{*}$ and $S^{*}$ are the restoring temperature and salinity, respectively; $\tau_{1,T}$ and $\tau_{1,S}$ are the restoring timescales for the surface temperature and salinity, respectively; and $\tau_{2}$ is the deep restoring timescale that is the same for temperature and salinity. Surface and deep temperature and surface salinity follow a seasonal trend where $A$ is the amplitude and $\varphi$ is the phase. The system is initially solved for the next time step giving values ($\hat{T_{1}}$, $\hat{T_{2}}$, $\hat{S_{1}}$, and $\hat{S_{2}}$). If the potential density from the surface box ρ_θ1_(*T*_1_*,S*_1_) is greater than that of the deep box, the two boxes completely and instantaneously mix such that:

$T_{1}= T_{2}=\frac{h_{1}\hat{T_{1}}+h_{2}\hat{T_{2}}}{h_{1}+h_{2}}$ Eq. (SI5)

$S_{1}=S_{2}=\frac{h_{1}\hat{S_{1}}+h_{2}\hat{S_{2}}}{h_{1}+h_{2}}$ Eq. (SI6).

In these equations *h*_1_ and *h*_2_ are the depths of the surface and deep boxes, respectively. This system of equations was fit to temperature and salinity data (NEFSC Oceanography Branch) representative of the study areas using the unconstrained optimization procedure described in Glover et al. [2]. The cost function was defined as the sum of the square of the differences between model and observations in potential temperature and salinity, weighted by the thermal and haline expansion coefficients [1]. The biogeochemistry of the surface and deep boxes is defined as:

$\frac{d{DIC}_{1}}{dt}= \left( J_{air}+K_{d}\frac{\partial DIC}{\partial z}-NCP-NCP*PICPOC \right)/h_{1}$ Eq. (SI7)

$\frac{d{DIC}_{2}}{dt}= \left( Remin*NCP+NCP*PICPOC-K_{d}\frac{\partial DIC}{\partial z} \right)/h_{2}$ Eq. (SI8)

$\frac{d{TA}_{1}}{dt}= \left( K_{d}\frac{\partial TA}{\partial z}-2*NCP*PICPOC \right)/h_{1}$ Eq. (SI9)

$\frac{d{TA}_{2}}{dt}= \left( -K_{d}\frac{\partial TA}{\partial z}+2*NCP*PICPOC \right)/h_{2}$ Eq. (SI10)

and mixed according to the same rules as Eqs. (SI5) and (SI6). In Eqs. (SI7) – (SI10), $J_{air}$ is the air-water CO_2_ flux such that $J_{air}=K_{air}\alpha_{{CO}_{2}}\left( {pCO}_{2,atm}-{pCO}_{2,surf} \right)$, $K_{air}$ is the gas transfer velocity calculated as in Wanninkhof [3] with a seasonally variable wind speed determined from the nearest NDBC monitoring buoy (GB, Station 44011; MA, Station 44009), $\alpha_{{CO}_{2}}$ is the CO_2_ solubility calculated as in Weiss [4], ${pCO}_{2,atm}$ is the atmospheric partial pressure of CO_2_, and ${pCO}_{2,surf}$ is the partial pressure of CO_2_ in the surface box (computed as a function of surface *T, S, DIC* and *TA* using CO2SYS for Matlab [5]).We define $K_{d}$ as the turbulent diffusion coefficient between the two boxes, and the vertical gradient ($\frac{\partial DIC}{\partial z}$) is approximated by the difference in concentration of the tracer from the center of each box. Here, $NCP$ is net community production such that $NCP=NPP*F_{ratio}$ where the seasonally variable net primary production (*NPP*) climatology was determined from ocean color [6] (<http://www.science.oregonstate.edu/ocean.productivity/index.php>), $PICPOC$ is the ratio of CaCO_3_ formed to particulate organic carbon produced from net community production, and $Remin$ is the organic matter remineralization efficiency in the deep box. All fluxes in Eqs. (SI7) – (SI10) are in units of mmol m^-2^ yr^-1^. The full carbonate chemistry in both boxes is then calculated using CO2SYS for Matlab with H_2_CO_3_ and HCO_3_^-^ dissociation coefficients from the Mehrbach refit and HSO_4_^-^ dissociation constant from Dickson and Millero [7]. Phosphate and silicate concentrations are assumed to be zero. Atmospheric CO_2_ has a sinusoidal seasonal trend approximated by the closest atmospheric CO_2_ station in Worcester, MA, USA (NOAA). Values for BGC model parameters are given in Table 3.

*Scallop model:*

The population at each time step, *t* (yr), is represented by a vector ***n***(*t*) (# of individuals) whose *h* elements indicate how many individuals are in each bin of the size class vector ***H***, which in this model consist of 5 mm shell length increments. The square matrix **G*_t_*** (unitless), where the columns of **G_t_** are the shell height bins of the current time step and the rows are the shell height bins of the next time step, contains sub-diagonal elements that represent the probability of growth into the new size class over one time step. When ***n***(*t*) is matrix multiplied by **G_t_** the resultant vector ***n***(*t*+∆*t*) indicates how many individuals are in each size group at the next time step and the complete population dynamics can be summarized by:

$\boldsymbol{n}\left( t+\Delta t \right)=R\Delta t+ \mathbf{G}_{\boldsymbol{t}}\boldsymbol{*}\boldsymbol{e}^{\boldsymbol{-(M+}\boldsymbol{Z}_{\boldsymbol{t}}\boldsymbol{)\Delta t}}\boldsymbol{*n}\mathbf{(}\boldsymbol{t)}$ Eq. (SI11)

where *R* (individuals yr^-1^) is the number of individuals that recruit into the first size bin ***n***(1). The exponential term is a diagonal matrix whose elements reflect the fraction of individuals remaining in the population during a time step ∆*t* after harvest and non-harvest mortalities ***Z*** (yr^-1^). Natural mortality (*M*, yr^-1^) also acts on the population. The scallop model time step ($\Delta t$) is 1/10 year. In the model section below, vectors over the size class bins (1 to *h*) are marked in bold and element-by-element scalar multiplication is implied for all vector operations except in Eq. SI11 where matrix multiplication is explicitly denoted by a *.

The probability distribution along the sub-diagonal of **G*_t_*** is based on estimated mean and variance in the von Bertalanffy growth parameters from observed growth increments in shells [8,9]. This distribution is calculated from geographically specific maximum lengths ($H_{\infty}, mm$) and Brody growth coefficients (*K*, yr^-1^) ([9] and Table 1 in [10]):

$H_{t+\Delta t}=H_{t}+\left( H_{\infty}-H_{t} \right)(1-e^{-K\Delta t})$ Eq. (SI12)

where *H_t_* and $H_{t+\Delta t}$ are the shell lengths in the current and next time steps, respectively. The probabilities in **G*_t_*** are determined from 10000 iterations at each shell height in 1 mm increments from 40mm to *H_∞_* of equation SI12 assuming $H_{\infty}$ and *K* have a gamma distribution [8,9]. **G*_t_*** is recalculated every time step *t* to accommodate links to the biogeochemical model (see above), since biogeochemical conditions can alter growth rates. Scallops “recruit”, or enter the model in the first size class (40-45 mm shell height), when they are about 2 years old.

This approach does not explicitly model spawning or scallop larval history before the scallops recruit at 40 mm, but differential growth rates will undoubtedly affect larval growth as well as post-recruit growth. Rather, Beverton-Holt stock-recruit relationships are used to determine annual recruitment, which has the form:

$R= \frac{\alpha_{R}SSB}{\gamma_{R}+SSB}$ Eq. (SI13)

where α_R_ is the recruitment asymptote (in millions), γ_R_ is the half saturation coefficient in metric tons (mT meats), and *SSB* is spawning stock biomass (mT meats). Interannual stochasticity in recruitment is included in the model by drawing $\alpha_{R}$ and $\gamma_{R}$ from a multivariate log-normal distribution [9]. The coefficients for this model are determined for each location through the NFMS Stock Assessment Workshops [9,10]. In a simplification of observed population dynamics, modeled recruitment is assumed to occur evenly throughout the year.

Scallops are subject to several types of mortality that contribute to total fishing mortality ***Z_t_*:**

$Z_{t}=\left\{ \begin{aligned} \left( I+LD \right)F_{t} H<90 \mathrm{mm} \\ \left( I+L \right)F_{t} H\geq90 \mathrm{mm} \end{aligned} \right.$ Eq. (SI14)

All scallops, regardless of size, are subject to incidental (non-catch, dredge-caused) mortality (*I* , yr^-1^) in addition to fishing mortality (*F_t_*, yr^-1^), as in Hart ([10], and references therein), which is set by the socioeconomic submodel. Incidental mortality scales with *F_t_*. Selectivity of the scallop gear increases with size according to a logistic selectivity vector ($\boldsymbol{L}$) described by Hart [9] and Hart et al. [12]:

$\boldsymbol{L}= \frac{1}{1+e^{a_{L}-b_{L}\boldsymbol{H}}}$ Eq. (SI15)

where $a_{L}$ and $b_{L}$ are fitting coefficients [10]. Scallops below 90 mm caught in the dredge are not kept, and they are subject to an additional discard mortality (*D*, yr^-1^) that also scales with *F_t_* [9]. All scallops above 90 mm are kept to contribute to the harvest.

Because the two submodels are integrated asynchronously, communicating only once a year on the socioeconomic model time step, approximations are made in the time integration of the scallop population submodel to ensure that the total catch and the landings removed from the population submodel are consistent with those from the socioeconomic submodel. At the end of each model year, the socioeconomic model computes a total catch for the next year using the end of year population biomass and allowable biological catch. That is, the allowable catch (*Q_ABC_*) changes yearly, but the maximum sustainable yield (*F_MSY_*) does not change. That catch is then removed uniformly over the time steps of the next year in the scallop submodel using the time-evolving size distribution to allocate catch by size bin.

A catch vector (***CA***, number of individuals caught per size class in a time step) is computed each time step based on the fraction of total mortality resulting from catch and the population distribution:

$\boldsymbol{CA}=\frac{\boldsymbol{F}_{\boldsymbol{t}}\boldsymbol{L}}{\boldsymbol{Z}_{\boldsymbol{t}}+M}\left( 1-e^{-\left( M+\boldsymbol{Z}_{\boldsymbol{t}} \right)\Delta t} \right)\boldsymbol{n}(t)$ Eq. (SI16)

Multiplying ***CA*** by a meat weight vector (***W***, g ind^-1^), converted to lb ind^-1^, and summing over the size class bins leads to the annualized catch rate:

$Q_{t}=\frac{1}{\Delta t}\sum_{i=1}^{h} CA\left( i \right)W(i)$ Eq. (SI17)

Meat weights are determined as a function of shell height through well-established relationships from [13]:

$\boldsymbol{W}=exp(a_{mw}+b_{mw}\ln\left( \boldsymbol{H} \right))$ Eq. (SI18)

where $a_{mw}$ and $b_{mw}$ are site-specific fitting coefficients (Table 1) and $\boldsymbol{H}$ is shell height. The final shell height bin of **G_t_** includes scallops that may be larger than *H_∞_*, thus it receives a special weight (Table 1). As the catch *Q_t_* specified from the socioeconomic submodel will not match exactly the catch determined in Eq. (SI17), the model iteratively solves for *F_t_* in Eq. (SI14) and (SI16) each time step such that Eq. (SI17) equals the *Q_t_*, specified from the socioeconomic submodel. Eqs. (SI1) – (SI7) are calculated separately for scallop populations in GB and MA, where the total catch (*Q_t_*) determined by the socio-economic submodel is separated into catch from GB and MA by assuming the landings extracted from each location are proportional to the exploitable biomass there (individuals > 90mm).

*Socioeconomic model:*

The socioeconomic submodel is based on a combination of approaches from 3 sources: 1) the New England Fisheries Management Council’s (current management practices of scallop industry) price and cost models [14–16], and their convention on industry behavior, 2) static economic theory from Moore [17], and 3) socioeconomic decision making from Nobre [18]. The socioeconomic model hinges on the development of a Cobb-Douglas production function using multiple linear regression, assuming that the total industry catch is related to the total scallop biomass and the number of days fished [19] such that:

$\ln\left( Q_{t} \right)=\ln\left( A \right)+\alpha\ln\left( \mathrm{BIO} \right)+\beta ln(DAS)$ Eq. (SI19)

where *Q_t_* is catch (lb yr^-1^, NMFS landings data), $BIO$ is total biomass (lb meats, [10]), $DAS$ is days fished for the whole fleet [14], and *A* (lb), $\alpha$, and $\beta$ are fitting coefficients (Table 2). Gross industry revenue (*GR*) is the product of *Q_t_* and the average price across all market categories:

${GR}_{t}=Q_{t}\bar{P_{i,t}}$, Eq. (SI20)

where $P_{i,t}$ is determined with the NEFMC [14] ex-vessel pricing model, which uses the previous year’s landings and the distribution of landings across market size categories *i* (where *i* is U10, 11-20, 21-30, or 31-40) to inform the current year’s scallop prices:

${ln(P}_{i,t})=0.704-0.00441{MCT}_{i}+0.132{IP}_{t}+0.0255{PCDI}_{t}-0.00131{LAND}_{net,t-1}-0.175{PCTLAND}_{i,t-1}+0.078U10+0.212D05+0.165D10$ . Eq. (SI21)

Here, $P_{i,t}$ is the price of scallops in market class $i$ for year $t$, ${MCT}_{i}$ is the mean scallop meat count in market class $i$, ${IP}_{t}$ is the price of imports in year $t$, ${PCDI}_{t}$ is per capita disposable income for year *t* for the United States corrected to 2011 dollars (US Bureau of Economic Analysis, “Table 2.1 Personal Income and its Disposition”, Accessed Oct. 10, 2013), ${LAND}_{net,t-1}$ is the difference of domestic landings *Q_t_* and exports in the previous year, ${PCTLAND}_{i,t-1}$ is the percentage of landings by weight (*Q_t_)* from market category $i$ in the previous year, $DU10$ is a dummy variable for a price premium for U10 scallops, and $D05$ and $D10$ are dummy variables to account for jumps in market prices in 2005 and 2010 due to increased international demand during those years. We do not attempt to predict price jumps for future years, and so the dummy variables simply improve the fit of this regression equation. If inflation is added to the scenarios, both ${IP}_{t}$ and ${PCDI}_{t}$ are assumed to increase at the rate of inflation; however, for this paper, inflation is not included. During model forecasts, scallop exports are determined by assuming the ratio of landings and exports remains constant and equal to the ratio of landings and exports in 2011.

Gross revenue is the sum of profits and fixed and operating costs. Gross revenues minus association fees ${(ASSN}_{t})$, communications costs $({COMM}_{t})$ [14], and a captain’s bonus of 5% of the revenues $(CAPT),$ is called “net stock” (*NS*), which is then split into boat share (*BS*) and crew share (*CS*) as per the current industry practice:

$NS=({GR}_{t}-{ASSN}_{t}-{COMM}_{t}-CAPT)$, where Eq. (SI22)

$BS=0.48(NS)$ , and Eq. (SI23)

$CS=NS-BS=0.52\left( NS \right)$. Eq. (SI24)

Profits [14] are determined from the boat share,

$GP=BS-{FC}_{t}$ , Eq. (SI25)

where vessel fixed costs (${FC}_{t}$) in year $t$ include average costs such as maintenance, repairs, engine and gear replacement, and hull and liability insurance, determined through NEFSC cost surveys from 2001-2007 [14], corrected by the consumer price index (CPI) from the US Bureau of Labor Statistics (BLS) to 2011 USD.

Net crew income ($NCI$) is determined from the crew share ($CS$) and industry costs (*C*, Equation 1):

$NCI=CS-C$ . Eq. (SI26)

*C* is calculated using a series of constants (Eq. 1) and *w*, determined from the following statistical relationship determined in the scallop Framework 22 [15]:

$w=3.991+0.286\ln\left( GRT \right)+0.632\ln\left( CREW \right)+0.843\ln\left( FUEL \right)-0.278DFT-0.399TRW$ Eq. (SI27)

where $GRT$ is the average gross tonnage from the vessel fleet; $CREW$ is the average number of crew per vessel, assumed to be 7 due to current regulations on crew size; $FUEL$ is the cost of fuel per gallon, which, although unrealistic, remains constant throughout model future projections in this manuscript to conform with the zero inflation rate also assumed in the model runs discussed in this paper.$DFT$ and $TRW$ are dummy variables that take the value of 1 for dredge or trawl gear. All vessels are assumed to use scallop dredges for harvest gear.

In the current management regime, fishing effort is limited by allocating the number of days at sea available to the fleet. The maximum allocated days at sea (*DAS_max_)* is calculated using NEFSC relationships for deriving landings per unit effort (*NLPUE*), which is the number of scallops caught per day at sea. This is modeled as a Holling-type II predator-prey relationship that depends on the exploitable number of scallops for the whole stock ($\bar{N}$) such that:

$NLPUE= \frac{43183\bar{N}}{\sqrt{{30626}^{2}+\bar{N}^{2}}}$ Eq. SI28

where $\bar{N}= \bar{\sum\boldsymbol{n}_{\boldsymbol{MA}}\left( \boldsymbol{t} \right)\boldsymbol{L}_{\boldsymbol{MA}}\boldsymbol{+}\sum\boldsymbol{n}_{\boldsymbol{GB}}\boldsymbol{(t)}\boldsymbol{L}_{\boldsymbol{GB}}}$ [20,21], ***n_MA_(t)*** and ***n_GB_(t)*** are the population vectors, ***L_MA_*** and ***L_GB_*** are the logistic selectivity vectors (*MA* and *GB* indicate subpopulations), and the overbars indicate averaging over the year. If the mean meat weight of exploitable scallops (g) is greater than 20 grams, the parameters in Eq. (SI28) are reduced by a factor of $\sqrt{\frac{20}{g}}$ which reflects that large scallops take longer to shuck [10,20,21]. The fishing mortality per vessel from a single day at sea [$F_{das}$, (day at sea)^-1^] is then calculated such that:

$F_{das}=\frac{NLPUE}{\bar{N}}$ Eq. (SI29)

The maximum days at sea per year (*DAS_max_*, (day at sea)/yr)^[[1]](#footnote-1)^ is determined as:

$F_{das}{DAS}_{max}= F_{msy}$ . Eq. (SI30)

Finally, *Q_ABC_* and *DAS_max_* are passed to the socioeconomic submodel to act as management-set upper limits when calculating the optimal catch (*Q_t_)*.

**Supplementary Information References:**

1. Rahmstorf S. A simple model of seasonal open ocean convection Part I: Theory. Ocean Dyn. 2001;52: 0026–0035. doi:10.1007/s10236-001-8174-4

2. Glover DM, Jenkins W, Doney SC. Modeling methods for marine science. Cambridge; New York: Cambridge University Press; 2011.

3. Wanninkhof R. Relationship between wind speed and gas exchange over the ocean. J Geophys Res Oceans. 1992;97: 7373–7382. doi:10.1029/92JC00188

4. Weiss RF. Carbon dioxide in water and seawater: the solubility of a non-ideal gas. Mar Chem. 1974;2: 203–215.

5. Van Heuven S, Pierrot D, Rae JWB, Lewis E, Wallace DWR. MATLAB Program Developed for CO2 System Calculations. ORNL/CDIAC-105b. [Internet]. Oak Ridge, Tennessee: Carbon Dioxide Information Analysis Center, U.S. Department of Energy; 2011. Available: doi: 10.3334/CDIAC/otg.CO2SYS_MATLAB_v1.1

6. Behrenfeld MJ, Falkowski PG. Photosynthetic rates derived from satellite-based chlorophyll concentration. Limnol Oceanogr. 1997;42: 1–20. doi:10.4319/lo.1997.42.1.0001

7. Dickson AG, Millero FJ. A comparison of the equilibrium constants for the dissociation of carbonic acid in seawater media. Deep-Sea Res Part Oceanogr Res Pap. 1987;34: 1733–1743.

8. Hart DR, Chute AS. Estimating von Bertalanffy growth parameters from growth increment data using a linear mixed-effects model, with an application to the sea scallop Placopecten magellanicus. Ices J Mar Sci. 2009;66: 2165–2175. doi:10.1093/icesjms/fsp188

9. Hart DR. Quantifying the tradeoff between precaution and yield in fishery reference points. Ices J Mar Sci. 2013;70: 591–603. doi:10.1093/icesjms/fss204

10. Northeast Fisheries Science Center. 50th Northeast Regional Stock Assessment Workshop (50th SAW): 50th SAW Assessment Report. 2010.

11. Hart DR. Yield- and biomass-per-recruit analysis for rotational fisheries, with an application to the Atlantic sea scallop (Placopecten magellanicus). Fish Bull. 2003;101: 44–57.

12. Hart DR, Jacobson LD, Tang J. To split or not to split: Assessment of Georges Bank sea scallops in the presence of marine protected areas. Fish Res. 2013;144: 74–83. doi:10.1016/j.fishres.2012.11.004

13. Hennen DR, Hart DR. Shell height-to-weight relationships for Atlantic sea scallops (Placopecten magellanicus) in offshore U.S. waters. J Shellfish Res. 2012;31: 1133–1144. doi:10.2983/035.031.0424

14. New England Fishery Management Council. Framework 24 to the Scallop FMP and Framework 29 to the Multispecies FMP. 2012.

15. New England Fishery Management Council. Final Framework 22 to the Scallop FMP. 2011.

16. New England Fishery Management Council. Framework adjustment 21 to the Atlantic sea scallop FMP, including an environmental assessment, regulatory impact review, regulatory flexibility analysis and stock assessment and fishery evaluation (SAFE) report. Newburyport, MA: New England Fisheries Management Council; 2010.

17. Moore CC. Welfare impacts of ocean acidification: An integrated assessment model of the US mollusk fishery. National Center for Environmental Economics: U.S. Environmental Protection Agency; 2011 Dec. Report No.: 11-06.

18. Nobre AM, Musango JK, de Wit MP, Ferreira JG. A dynamic ecological-economic modeling approach for aquaculture management. Ecol Econ. 2009;68: 3007–3017. doi:10.1016/j.ecolecon.2009.06.019

19. Edwards SF. Rent-seeking and property rights formation in the U.S. Atlantic sea scallop fishery. Mar Resour Econ. 2001;16. Available: http://ideas.repec.org/a/ags/mareec/28123.html

20. Northeast Fisheries Science Center. 45th Northeast Regional Stock Assessment Workshop (45th SAW): 45th SAW Assessment Report. 2007. Report No.: NEFSC Ref Doc. 07-16.

21. Valderrama D, Anderson JL. Improving utilization of the Atlantic sea scallop resource: An analysis of rotational management of fishing grounds: Reply. Land Econ. 2009;85: 383–389.

1. We have chosen to use *F_msy_* instead of *F_ABC_* because the economic relationship for estimating operating costs is based on Vessel Trip Report (VTR) data. *DAS_max_* is days charged from the Vessel Monitoring System (AMS)’s demarcation line, whereas VTR-measured days from port is somewhat greater than that of AMS data. If we use *F_ABC_* we will likely underestimate landings. [↑](#footnote-ref-1)
